# Ocrelizumab exposure in relapsing–remitting multiple sclerosis: 10-year analysis of the phase 2 randomized clinical trial and its extension

**DOI:** 10.1007/s00415-023-11943-4

**Published:** 2023-10-31

**Authors:** Ludwig Kappos, Anthony Traboulsee, David K. B. Li, Amit Bar-Or, Frederik Barkhof, Xavier Montalban, David Leppert, Anna Baldinotti, Hans-Martin Schneble, Harold Koendgen, Annette Sauter, Qing Wang, Stephen L. Hauser

**Affiliations:** 1 Research Center for Clinical Neuroimmunology and Neuroscience Basel (RC2NB), University Hospital and University of Basel, Basel, Switzerland; 2 Department of Medicine (Neurology), University of British Columbia, Vancouver, BC Canada; 3 Department of Radiology and Medicine (Neurology), University of British Columbia, Vancouver, BC Canada; 4 Center for Neuroinflammation and Experimental Therapeutics, and Department of Neurology, Perelman School of Medicine, University of Pennsylvania, Philadelphia, PA USA; 5 VU University Medical Centre, Amsterdam, The Netherlands; 6 UCL Institutes of Biomedical Engineering and Neurology, London, UK; 7 Department of Neurology-Neuroimmunology, Centre d’Esclerosi Múltiple de Catalunya (Cemcat), Hospital Universitari Vall d’Hebron, Barcelona, Spain; 8 Departments of Medicine, Biomedicine and Clinical Research, University Hospital Basel, University of Basel, Basel, Switzerland; 9 F. Hoffmann-La Roche Ltd, Basel, Switzerland; 10 UCSF Weill Institute for Neurosciences, Department of Neurology, University of California San Francisco, San Francisco, CA USA; 11 Present Address: UCB Farchim SA, Bulle, Switzerland; 12 Present Address: Janssen Pharmaceuticals, Allschwil, Basel-Landschaft Switzerland

**Keywords:** Ocrelizumab, Multiple sclerosis, Disease-modifying therapies, Safety

## Abstract

Open-label extension (OLE) studies help inform long-term safety and efficacy of disease-modifying therapies in multiple sclerosis (MS). We report exploratory analyses from a phase 2 trial on the longest follow-up to date of ocrelizumab-treated patients with relapsing–remitting MS (RRMS). The primary treatment period (PTP) comprised four 24-week treatment cycles; participants were randomized to double-blind ocrelizumab (2000 mg or 600 mg), placebo, or interferon β-1a (open label) for one cycle, then dose-blinded ocrelizumab 1000 mg or 600 mg for the remaining cycles. The PTP was followed by consecutive assessed and unassessed treatment-free periods (TFPs) and then the OLE (ocrelizumab 600 mg every 24 weeks). Safety and efficacy were prospectively assessed. Of 220 participants randomized, 183 (84%) completed the PTP. After the TFP, 103 entered OLE (median OLE ocrelizumab exposure 6.5 years). Most common adverse events across all periods were infusion-related reactions. MRI activity, annualized relapse rate, and confirmed disability progression (CDP) rates remained low throughout. During the assessed TFP, there was a trend toward less and later B-cell repletion, and later CDP, for patients randomized to ocrelizumab; MRI activity was observed in 16.3% of patients, the earliest 24 weeks after the last ocrelizumab dose. This is the longest follow-up of ocrelizumab-treated patients with RRMS, with no new safety signals emerging during an observation period from 2008 to 2020. Results reinforce the sustained efficacy of long-term ocrelizumab. Reduced disease activity was maintained following interruption of 6-month dosing cycles, with no evidence of rebound.

**Supplementary Information:**

The online version contains supplementary material available at 10.1007/s00415-023-11943-4.

## Introduction

Ocrelizumab (Ocrevus, Genentech, Inc., California, USA) is an anti-CD20 monoclonal antibody approved for the treatment of relapsing and primary progressive multiple sclerosis (RMS/PPMS). Phase 3 data showed significant benefit in clinical (confirmed disability progression, annualized relapse rate) and MRI measures with sustained efficacy in the open-label extension (OLE), where adverse events (AEs) were consistent with past reports and no new safety signals emerged with prolonged treatment [1, 2]. Given the life-long condition of MS with a high risk of irreversible disability over time, and considering the potential risks of long-term treatment, the collection of long-term safety and efficacy outcomes is important to inform the patient–clinician dialogue in treatment choices.

Here we present long-term follow-up data from the phase 2 study of ocrelizumab in relapsing–remitting MS (RRMS) (NCT00676715). This study was a multicenter, randomized, parallel-group, placebo-, and interferon (IFN) β-1a–controlled dose-finding study designed to evaluate the efficacy and safety of two dose regimens of ocrelizumab in patients with RRMS. The study was originally designed with a treatment duration of 96 weeks (primary treatment period [PTP]) followed by a treatment-free safety follow-up period (TFP) of at least 48 weeks with the main aim of assessing B-cell repletion. After the favorable primary study readout, an OLE was added to assess long-term safety and efficacy.

The primary endpoint at week 24 showed a highly significant reduction in gadolinium (Gd)-enhancing brain MRI lesions with ocrelizumab treatment vs placebo; the number of Gd-enhancing lesions was 89% (*p* < 0.0001) lower in the 600 mg ocrelizumab group than in the placebo group, and 96% (*p* < 0.0001) lower in the 2000 mg group [3]. Patients subsequently continued on ocrelizumab and had their last PTP assessment at week 96 to account for the treatment effect from the last 6-monthly dose (at study week 72) before entering the TFP.

We present herein a post hoc analysis of safety and efficacy for the PTP, the TFP, and the OLE through January 2020, covering an observation period of more than a decade.

## Methods

### Study design and participants

The original phase 2 study design and procedures have been fully described previously, together with the primary and key secondary endpoints through treatment week 48 [3]. Briefly, the study recruited adults (18–55 years) with RRMS [4] who had at least two relapses within 3 years of screening (one or more within 1 year), had a baseline Expanded Disability Status Scale (EDSS) score [5] of 1–6, and had evidence of recent MS activity defined as at least six T2 lesions on a MRI scan done in the year prior to screening or two relapses within the year prior to screening. Participants were recruited at 79 centers in 20 countries from North America, Latin America, East-central Europe, and Western Europe, and randomized to four treatment groups via an interactive voice recognition service, stratified by geographic region.

**Fig. 1:**
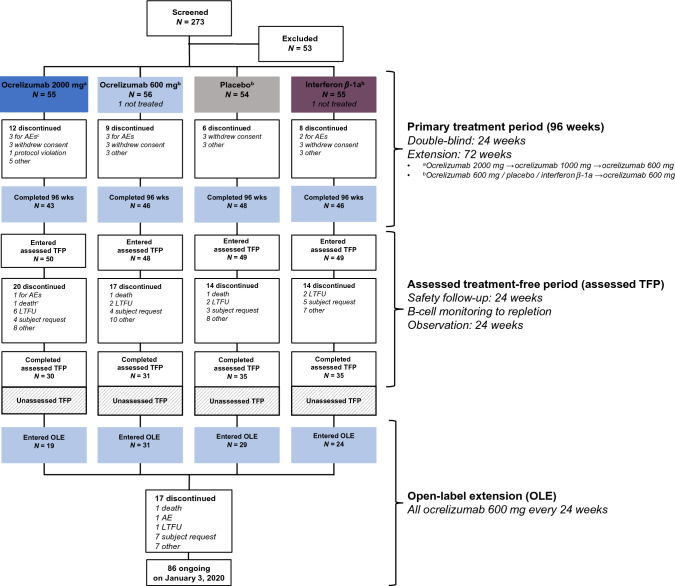
Patient disposition. Treatment assignments refer to cycle 1 dosing only. All patients received ocrelizumab in treatment cycles 2–4. Patients discontinuing the PTP were asked to enter the assessed TFP. *AE* adverse event, *assessed TFP* assessed treatment-free period, *LTFU* lost to follow-up, *OLE* open-label extension, *PTP* primary treatment period, *wk* week. ^c^Under study statistical coding rules, one patient who died on study day 92 from systemic inflammatory response syndrome was classified as having withdrawn from the PTP for an AE from which the patient subsequently died in the assessed TFP

The study consisted of four periods: a 96-week PTP, a TFP consisting of a preplanned assessed TFP of at least 48 weeks for all patients and followed for most patients by a variable period where patients were not assessed (unassessed TFP). Patients who had completed the assessed TFP were offered subsequent entry into the OLE (see Supplemental Material for additional details of study periods). The duration of the assessed TFP varied because patients were followed at 12-week intervals until B cells were repleted. Both the repletion-dependent variable duration of the assessed TFP and the late addition of the OLE to the protocol resulted in variation of the duration of the unassessed TFP. A study flow diagram, schematic of the study design, and duration of the study TFP for each patient who entered the OLE to receive ocrelizumab 600 mg every 24 weeks are shown in Fig. 1 and Supplementary Figs. 1 and 2, respectively.

### Randomization and masking

Details of randomization and masking in the PTP have been reported previously [3].

### Objectives

The objective of these exploratory analyses was to report long-term safety and efficacy of ocrelizumab treatment in RRMS (see Supplemental Material for the details of procedures).

### Outcomes

The previously reported [3] primary study endpoint was the total number of Gd-enhancing T1 lesions observed on brain MRI scans from week 12 to week 24 (cycle 1) for those receiving ocrelizumab vs placebo.

Secondary and exploratory MRI-derived endpoints included the total number of Gd-enhancing T1 lesions for all data points in cycle 1; total number of new Gd-enhancing T1 lesions; the number of new or enlarging T2 lesions compared with the previous MRI time point; and brain parenchymal fraction (BPF) [6].

Brain volume and its change were assessed as an exploratory MRI endpoint at weeks 0, 12, and 96 of the PTP in those randomized to receive ocrelizumab in cycle 1, and at OLE baseline and OLE week 96 in all OLE participants.

Clinical outcomes collected during PTP, assessed TFP, and OLE included the annualized protocol-defined relapse rate (ARR) and confirmed disability progression (CDP) on the EDSS [5, 7].

Safety evaluations included regular neurologic and physical examinations, vital signs, electrocardiographs, and the occurrence of AEs. AEs were classified according to the Medical Dictionary for Regulatory Activities (MedDRA v22.1) and graded per Common Terminology Criteria version 3.0 and were reported as rates per 100 patient years (PY) to account for differing durations of exposure to ocrelizumab. Categories of interest for AEs included fatal cases, infections, infusion-related reactions, and malignancies.

Laboratory assessments including CD19+ B-cell and T-cell counts, and immunoglobulin concentrations (IgA, IgG, IgM) were performed. CD3+, CD3+ CD4+, and CD3 CD8+ T cells were also measured. Mature naive B cells could not be reported due to a technical issue with the flow cytometry panel.

### Statistical procedures

The primary analysis and rationale for the number of participants enrolled have been published previously [3]. The data cutoff for analyses of the ongoing OLE was January 3, 2020. The presented analyses are post hoc, descriptive, and were not powered to detect statistically significant differences in safety or efficacy outcomes among different treatment groups.

Safety and efficacy data are presented by randomization group for the PTP and assessed TFP, and for all participants in the OLE irrespective of initial study randomization.

To account for differing exposure lengths, overall AEs, serious AEs (SAEs), overall infections, and serious infections are reported as rates per 100 PY. Rare serious events (death, malignancy) are listed separately. Infusion-related reactions are reported overall and by individual infusion in the PTP and the OLE.

The ARR was assessed by Poisson regression, adjusted for geographic region and offsetting for exposure time in years. ARR data were assessed overall and by treatment cycle in the 96-week PTP, and overall, for the OLE up to data cutoff. Time to CDP of at least 24 weeks (24WCDP) and to B-cell repletion were assessed by Kaplan–Meier analysis. Treatment differences were assessed by log-rank test stratified by region.

## Results

Patients were randomized between July 17, 2008, and April 1, 2009. Of 273 participants screened, 220 were randomized and 218 were treated, of whom 183 (84%) completed all four cycles (96 weeks) in the PTP. Including participants who had discontinued treatment early, 196 (90%) entered the assessed TFP—of whom 131 (60% of the 220 originally randomized) completed this period. Of these, 103 (47% of the 220 originally randomized) subsequently entered the OLE. At time of data cutoff, 86 (83%) of the 103 OLE participants (39% of those originally randomized) remained on treatment. Disposition through the three periods is shown in Fig. 1.

Baseline characteristics at the start of the PTP were similar between randomization groups, and, with the exception of disease duration at OLE entry, OLE baseline characteristics for the 103 participants were similar to the all-patient characteristics at study baseline (Supplementary Table 1). Similarly, study baseline characteristics for those who did or did not enter the OLE were broadly similar (Supplementary Table 1).

The median overall duration of the TFP was 92.6 weeks (interquartile range [IQR] 60.8–131.0). The median duration of the assessed TFP was 61.0 weeks (IQR 49.9–88.6). This aggregate duration was comparable between those who did and those who did not subsequently enter the OLE (median [IQR] 61.7 [51.1–95.7] vs 60.0 [49.0–87.1]) weeks, respectively. For the 103 participants in the OLE, the median length of the unassessed TFP following completion of the assessed TFP was 35.9 weeks (IQR 20.9–65.6 weeks).

For each of the 103 OLE participants, individual treatment-free durations (assessed TFP plus unassessed TFP) from the end of PTP to the start of the OLE are shown in Supplementary Fig. 2.

The median duration of OLE treatment at time of data cutoff was 6.5 years (338 weeks); IQR was 316.0–361.7 weeks. Most OLE participants (89/103; 86%) had reached at least week 240 (4.6 years).

Exposure-adjusted AE rates, SAE rates, and the most common AEs are shown in Table 1. Exposure-adjusted rates of overall AEs, SAEs, and infections (including serious infections) were broadly comparable across the four randomized groups in the PTP. Overall AEs and infections were slightly lower numerically in the IFN β-1a randomized group; however, the rate of SAEs was numerically higher in the IFN β-1a arm. Overall, AE rates were lower in the assessed TFP than in the PTP. The adjusted rate of serious infections was low in participants receiving ocrelizumab in the PTP and remained low in the OLE, while exposure-adjusted rates of overall AEs and infections were numerically lower in the OLE than among participants receiving ocrelizumab during the PTP.

**Table 1:** Adverse event incidence rates per 100 patient years and incidence of most common adverse events per study period

	Primary treatment period (96 weeks)				Assessed treatment-free period				OLE
	Ocrelizumab 2000 mg *n* = 55	Ocrelizumab 600 mg *n* = 55	Placebo *n* = 54	Interferon β-1a *n* = 54	Ocrelizumab 2000 mg *n* = 50	Ocrelizumab 600 mg *n* = 48	Placebo *n* = 49	Interferon β-1a *n* = 49	Ocrelizumab 600 mg *n* = 103
**AE incidence rates per 100 patient years + 95% CI**									
Any AE	370.1 (331.9, 412.6)	337.0 (301.4, 376.7)	343.4 (308.3, 382.5)	281.4 (249.1, 317.9)	186.4 (157.3, 220.7)	75.6 (58.0, 98.5)	90.8 (70.3, 117.1)	69.9 (52.3, 93.3)	112.2 (104.1, 120.8)
SAE	8.0 (3.8, 16.8)	7.6 (3.6, 16.0)	6.2 (2.8, 13.9)	13.1 (7.4, 23.1)	7.0 (2.9, 16.7)	5.5 (2.1, 14.7)	1.5 (0.2, 10.9)	3.0 (0.8, 12.1)	5.3 (3.8, 7.4)
Infections	81.1 (64.3, 102.3)	87.2 (70.1, 108.6)	87.1 (70.4, 107.9)	58.9 (45.1, 76.9)	34.8 (23.5, 51.5)	23.4 (14.5, 37.6)	33.8 (22.3, 51.4)	21.3 (12.6, 35.9)	42.6 (37.8, 48.1)
Serious infections	2.3 (0.6, 9.1)	3.3 (1.1, 10.1)	5.2 (2.2, 12.5)	3.3 (1.1, 10.2)	0	0	0	1.5 (0.2, 10.8)	2.9 (1.8, 4.6)
**AEs occurring in > 10% of patients in any group in any period,** *n* **(%)**^**a**^									
Infusion-related reaction	27 (49.1)	22 (40.0)	26 (48.1)	16 (29.6)	NA	NA	NA	NA	23 (22.3)
MS relapse	11 (20.0)	15 (27.3)	22 (40.7)	25 (46.3)	16 (32.0)	9 (18.8)	6 (12.2)	6 (12.2)	26 (25.2)
Nasopharyngitis	9 (16.4)	8 (14.5)	6 (11.1)	6 (11.1)	6 (12.0)	2 (4.2)	3 (6.1)	4 (8.2)	18 (17.5)
Urinary tract infection	7 (12.7)	5 (9.1)	9 (16.7)	7 (13.0)	3 (6.0)	0	2 (4.1)	0	21 (20.4)
Upper respiratory tract infection	8 (14.5)	9 (16.4)	6 (11.1)	4 (7.4)	1 (2.0)	2 (4.2)	3 (6.1)	1 (2.0)	14 (13.6)
Respiratory tract infection	2 (3.6)	4 (7.3)	6 (11.1)	1 (1.9)	0	0	1 (2.0)	0	6 (5.8)
Headache	9 (16.4)	7 (12.7)	10 (18.5)	9 (16.7)	3 (6.0)	0	1 (2.0)	1 (2.0)	7 (6.8)
Back pain	4 (7.3)	4 (7.3)	4 (7.4)	6 (11.1)	0	1 (2.1)	4 (8.2)	1 (2.0)	6 (5.8)
Fatigue	8 (14.5)	3 (5.5)	2 (3.7)	4 (7.4)	1 (2.0)	1 (2.1)	1 (2.0)	0	8 (7.8)
Insomnia	8 (14.5)	2 (3.6)	1 (1.9)	1 (1.9)	2 (4.0)	0	0	0	4 (3.9)
**SAEs occurring in patients in any group in any period,** *n* **(%)**^**a**^									
Total number of patients with at least one serious adverse event	6 (10.9)	4 (7.3)	3 (5.6)	10 (18.5)	5 (10.0)	4 (8.3)	1 (2.0)	2 (4.1)	14 (13.6)
Overall total number of events	7	5	3	11	5	4	1	2	27
Anal abscess	0	1 (1.8)	0	0	0	0	0	0	0
Cystitis	0	1 (1.8)	0	0	0	0	0	0	0
Diverticulitis	0	0	0	1 (1.9)	0	0	0	0	0
Encephalitis	0	0	0	1 (1.9)	0	0	0	0	0
Gastroenteritis	0	0	1 (1.9)	0	0	0	0	0	0
Gingivitis	1 (1.8)	0	0	0	0	0	0	0	0
Influenza	0	0	0	1 (1.9)	0	0	0	0	0
Oral herpes	0	0	1 (1.9)	0	0	0	0	0	0
Pyelonephritis	1 (1.8)	0	0	0	0	0	0	0	0
Bronchitis	0	0	0	0	0	0	0	1 (2.0)	1 (1.0)
Pneumonia	0	0	0	0	0	0	0	0	2 (1.9)
Urinary tract infection	0	0	0	0	0	0	0	0	2 (1.9)
Cellulitis	0	0	0	0	0	0	0	0	1 (1.0)
Hepatitis A	0	0	0	0	0	0	0	0	1 (1.0)
Infection	0	0	0	0	0	0	0	0	1 (1.0)
Infective exacerbation of chronic obstructive airways disease	0	0	0	0	0	0	0	0	1 (1.0)
Sepsis	0	0	0	0	0	0	0	0	1 (1.0)
Tracheobronchitis	0	0	0	0	0	0	0	0	1 (1.0)
MS relapse	0	0	0	2 (3.7)	0	0	0	0	0
Epilepsy	1 (1.8)	0	0	0	0	0	0	0	0
Muscle spasticity	0	1 (1.8)	0	0	0	0	0	0	0
Seizure	0	0	0	1 (1.9)	0	0	0	0	0
Uhthoff’s phenomenon	0	0	0	0	0	0	0	0	1 (1.0)
Upper abdominal pain	0	0	1 (1.9)	0	0	0	0	0	0
Colitis	0	1 (1.8)	0	0	0	0	0	0	0
Strangulated inguinal hernia	0	0	0	1 (1.9)	0	0	0	0	0
Large intestine polyp	1 (1.8)	0	0	0	0	0	0	0	0
Salivary duct inflammation	0	0	0	0	0	1 (2.1)	0	0	0
Subileus	0	0	0	0	0	1 (2.1)	0	0	0
Anxiety	1 (1.8)	0	0	0	0	0	0	0	0
Suspected suicide attempt	0	0	0	1 (1.9)	0	0	0	0	0
Acute psychosis	0	0	0	0	1 (2.0)	0	0	0	0
Suicidal ideation	0	0	0	0	1 (2.0)	0	0	0	0
Retinal artery occlusion	0	0	0	1 (1.9)	0	0	0	0	0
Systemic inflammatory response syndrome	1 (1.8)	0	0	0	0	0	0	0	0
Death	0	0	0	0	0	1 (2.1)	0	0	0
Drug withdrawal syndrome	0	0	0	0	0	0	0	1 (2.0)	0
Pyrexia	0	0	0	0	0	0	0	0	1 (1.0)
Hypersensitivity	0	1 (1.8)	0	0	0	0	0	0	0
Infusion-related reaction	0	0	0	1 (1.9)	0	0	0	0	0
Fall	0	0	0	0	1 (2.0)	0	0	0	0
Injury	0	0	0	0	0	0	1 (2.0)	0	0
Subdural hematoma	0	0	0	0	0	0	0	0	1 (1.0)
Ureterolithiasis	1 (1.8)	0	0	0	0	0	0	0	0
Ovarian mass	0	0	0	1 (1.9)	0	0	0	0	0
Uterine prolapse	0	0	0	0	0	0	0	0	1 (1.0)
Immune thrombocytopenic purpura	0	0	0	0	1 (2.0)	0	0	0	0
Breast cancer	0	0	0	0	1 (2.0)	0	0	0	0
Pregnancy	0	0	0	0	0	1 (2.1)	0	0	0
Back pain	0	0	0	0	0	0	0	0	1 (1.0)
Rotator cuff syndrome	0	0	0	0	0	0	0	0	1 (1.0)
Anemia	0	0	0	0	0	0	0	0	1 (1.0)
Amylase increased	0	0	0	0	0	0	0	0	1 (1.0)
Blood potassium increased	0	0	0	0	0	0	0	0	1 (1.0)
Erythema nodosum	0	0	0	0	0	0	0	0	1 (1.0)
Hypertension	0	0	0	0	0	0	0	0	1 (1.0)

*AE* adverse event, *CI* confidence interval, *IFN* interferon, *MS* multiple sclerosis, *NA* not applicable, *OLE* open-label extension, *SAE* serious adverse event

^a^Table of most common events excludes influenza-like illness reported during the primary treatment period only in 11 patients (20.4%) who received IFN β-1a. Doses in primary treatment and treatment-free periods refer to cycle 1 randomization. All patients received ocrelizumab in cycles 2–4 of the primary treatment period

The most common AEs across all study periods were infusion-related reactions, infections, headache, and back pain. Other than the absence of infusion-related reactions in the assessed TFP, there was no change in the type of AEs seen across the three study periods. Infusion-related reactions occurred for the first infusion of ocrelizumab received in the PTP or in the OLE following an extended period off-treatment and were rarely observed after the fourth infusion (Supplementary Fig. 3). A higher rate of infections and serious infections was observed in the OLE compared with the TFP. There were no cases of progressive multifocal leukoencephalopathy (PML) in any study period. Details on malignancies and deaths are reported in Supplemental Material.

**Fig. 2:**
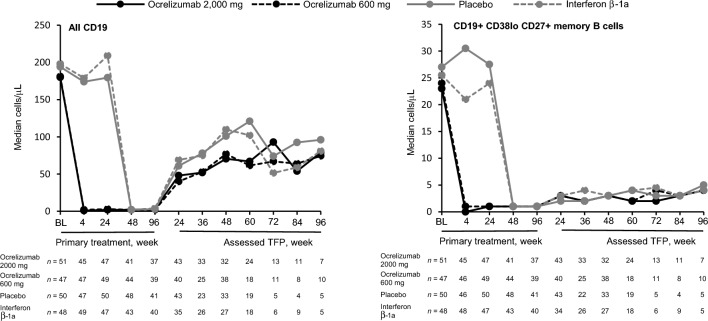
Median overall CD19 cell and CD19+ CD38lo CD27+ memory B-cell counts during the primary treatment and assessed treatment-free periods, by initial randomization group. From week 24 to week 96 of the PTP, all patients were on ocrelizumab. Almost all post-baseline medians for predose CD19 in the OLE (OCR 600 mg) were < 5 cells/µL and are not shown. *Assessed TFP* assessed treatment-free period, *BL* baseline, *OCR* ocrelizumab, *OLE* open-label extension, *PTP* primary treatment period

Overall blood CD19 cell counts and CD19+ CD38lo CD27+ memory B cells declined rapidly following ocrelizumab treatment in the PTP; both increased during the TFP again (Fig. 2). At OLE baseline, median (range) number of overall CD19+ and CD19+ CD38lo CD27+ memory B cells was 204.0 cells/µL (6.0–646.0) and 5.0 cells/µL (1.0–35.0), respectively. Post-baseline medians for both overall CD19 and CD19+ CD38lo CD27+ memory B cells in the OLE remained mostly below detection (data not shown), and plasmablast/plasma cell medians were reduced or undetectable at all time points in all three periods (data not shown).

For a repletion threshold of 80 CD19+ cells/µL, a trend was observed toward a longer time to repletion in the assessed TFP for participants randomized to receive ocrelizumab compared with the comparator groups who received ocrelizumab from cycle 2; however, the number of patients was small, and this trend was not evident for a repletion threshold of 40 cells/µL (Supplementary Fig. 4). Median (95% confidence interval [CI]) number of weeks to repletion with a threshold of 80 cells/µL was 74.0 (60.3–90.0) in the ocrelizumab 2000 mg randomization group (*n* = 55) and 71.9 (59.1–85.4) in the ocrelizumab 600 mg randomization group (*n* = 51), vs 62.0 (59.7–73.0) for the placebo randomization group (*n* = 51) and 59.0 (49.7–70.0) for the IFN β-1a randomization group (*n* = 49). For the 40 cells/µL threshold, these values were 50.6 (48.3–60.0) and 53.0 (48.0–59.7), vs 49.0 (48.0–50.1) and 49.9 (45.6–59.1), respectively.

Overall CD3+ T cells, CD3+ CD4+ T cells, and CD3+ CD8+ T cells remained stable during the PTP, assessed TFP, and OLE, with no apparent effect of initial randomization or subsequent ocrelizumab dose (Supplementary Fig. 5).

**Fig. 3:**
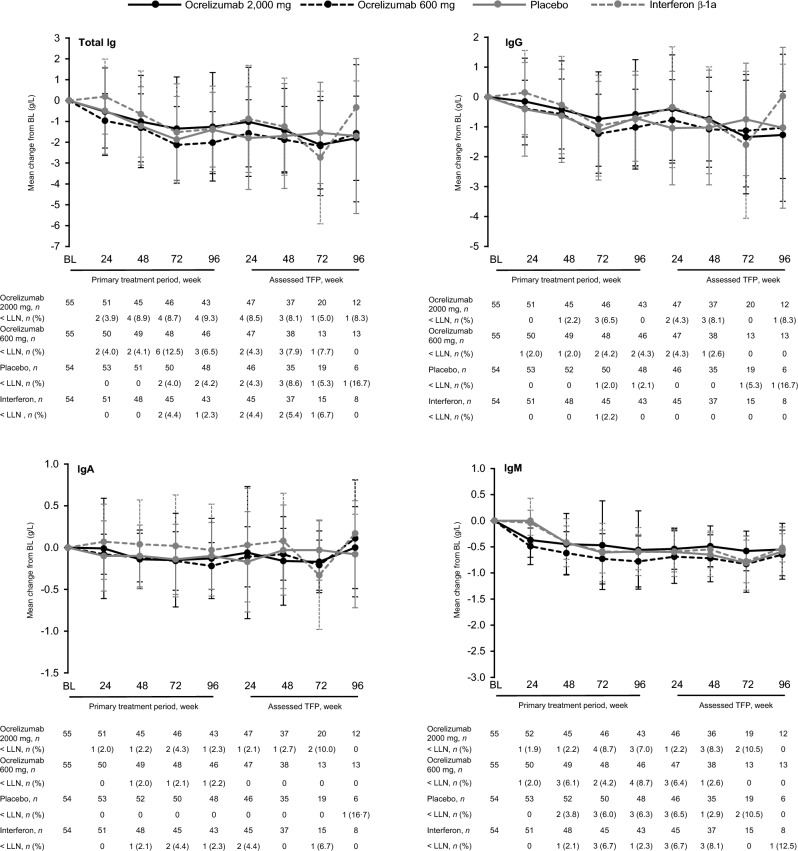
Mean (SD) changes from baseline in immunoglobulin (Ig) fractions in the primary treatment and post-treatment follow-up periods, by initial randomization group. *Assessed TFP* assessed treatment-free period, *BL* baseline, *Ig* immunoglobulin, *LLN* lower limit of normal.

Total Ig levels declined during the PTP, driven primarily by reductions in IgG, and remained relatively stable during the assessed TFP (Fig. 3). Furthermore, steady and ongoing declines in all Ig fractions were observed across the OLE (Supplementary Fig. 6), consistent with 6-year OLE data from the phase 3 studies of ocrelizumab [8].

**Table 2:** Brain MRI endpoints: number of gadolinium-enhancing T1 lesions and number of new and/or enlarging T2 lesions by visit and initial randomization group

	Total Gd-enhancing T1 lesions				New/enlarging T2 lesions			
	Ocrelizumab 2000 mg *n* = 55	Ocrelizumab 600 mg *n* = 55	Placebo *n* = 54	Interferon β-1a *n* = 54	Ocrelizumab 2000 mg *n* = 55	Ocrelizumab 600 mg *n* = 55	Placebo *n* = 54	Ιnterferon β-1a *n* = 54
Wk 0	2.23 (6.33) [53]	3.92 (9.88) [51]	1.64 (4.05) [47]	2.28 (5.26) [50]	–	–	–	–
Wk 4	1.52 (4.81) [46]	1.44 (3.41) [48]	1.12 (1.92) [50]	1.60 (3.13) [47]	1.02 (3.28) [46]	0.69 (1.89) [48]	0.98 (1.56) [50]	0.96 (2.13) [47]
Wk 8	0.22 (0.66) [46]	0.56 (1.05) [48]	1.96 (3.98) [49]	1.73 (3.62) [48]	0.07 (0.25) [46]	0.17 (0.48) [48]	1.47 (3.27) [49]	1.25 (2.77) [48]
Wk 12	0.05 (0.21) [44]	0.26 (0.68) [46]	1.31 (3.20) [42]	1.76 (4.01) [45]	0.00 (0.00) [44]	0.02 (0.15) [46]	1.02 (2.37) [42]	1.22 (2.87) [45]
Wk 16	0.02 (0.15) [45]	0.16 (0.43) [43]	0.83 (2.28) [41]	1.65 (3.50) [48]	0.00 (0.00) [45]	0.02 (0.15) [43]	0.83 (1.63) [41]	1.23 (2.75) [48]
Wk 20	0.05 (0.22) [40]	0.13 (0.41) [39]	1.56 (4.10) [43]	0.83 (2.11) [41]	0.00 (0.00) [40]	0.03 (0.16) [39]	1.14 (3.08) [43]	0.59 (1.82) [41]
Wk 24	0.05 (0.30) [44]	0.04 (0.21) [46]	1.36 (4.03) [47]	2.48 (6.06) [44]	0.00 (0.00) [44]	0.00 (0.00) [46]	1.43 (3.54) [47]	2.07 (5.62) [44]
Wk 96	0.00 (0.00) [43]	0.00 (0.00) [44]	ND	ND	0.07 (0.34) [43]	0.00 (0.00) [44]	ND	ND
Wk 144 (assessed TFP Wk 48)	0.32 (1.89) [34]	0.00 (0.00) [36]	ND	ND	1.03 (5.50) [34]	0.00 (0.00) [36]	ND	ND
OLE Wk 0^a^	0.13 (1.12) [98]				0.80 (3.48) [98]			
OLE Wk 96	0.00 (0.00) [91]				0.38 (1.61) [91]			

Data are mean (SD) [*n*]

*Gd* gadolinium, *ND* not determined, *OLE* open-label extension (600 mg ocrelizumab every 24 weeks), *TFP* assessed treatment-free period, *Wk* week

^a^Last evaluable off-treatment MRI before entering the OLE. Comprises week 144 data (*n* = 45) or OLE screening data (*n* = 53) where week 144 data were not available

**Fig. 4:**
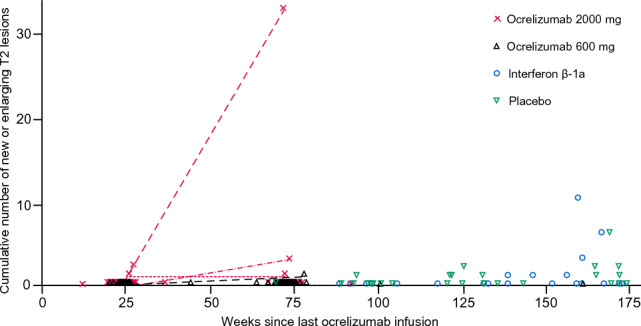
Cumulative number of new or enlarging T2 lesions by treatment after last ocrelizumab infusion up to and including the OLE baseline. Patients included had a 24-week MRI assessment and received at least three cycles of ocrelizumab during PTP. Participants randomized at baseline to receive ocrelizumab 2000 mg or 600 mg had scans at week 96 (i.e. 24 weeks after the last ocrelizumab treatment) and at week 144; other patients received an MRI at baseline OLE, which was compared with the 24-week MRI. Dots show all assessments performed; lines connect patients with more than one MRI assessment. One patient (randomized to the ocrelizumab 2000 mg group) had 32 new or enlarging T2 lesions and 11 T1 Gd-enhancing lesions 71 weeks after the last ocrelizumab infusion. This patient had three relapses during the assessed TFP (a clinical relapse at 67 weeks, and two protocol-defined relapses at 94 and 126 weeks, respectively, after the last ocrelizumab infusion). One patient (randomized to the ocrelizumab 600 mg group) had one new or enlarging T2 lesion and one T1 Gd-enhancing lesion at 78 weeks since last ocrelizumab infusion. One patient (randomized to the placebo group) had two new or enlarging T2 lesions and one T1 Gd-enhancing lesion at week 171 since last ocrelizumab infusion. *Assessed TFP* assessed treatment-free period, *Gd* gadolinium, *OLE* open-label extension

The mean number of observed Gd-enhancing T1 lesions during the first treatment cycle fell by 98–99% between baseline and week 24 among participants receiving ocrelizumab, compared with little or no reduction among those receiving placebo or IFN β-1a (Table 2). Among those randomized to ocrelizumab in the first cycle, mean Gd-enhancing T1 lesions continued to decline to 0 by end of PTP at week 96, and this decline was mostly maintained throughout the subsequent assessed TFP and on into the OLE. Only three of 141 participants (2%) from all four randomization groups with at least one post-ocrelizumab–treatment MRI assessment displayed Gd-enhancing T1 lesions during the assessed TFP: two had one lesion each (placebo/ocrelizumab 600 mg), and one had 11 lesions (ocrelizumab 2000 mg). There were three participants with Gd-enhancing lesions at OLE baseline. No Gd-enhancing T1 lesions were noted in any participant at OLE week 96 (Table 2). For the number of new or enlarging T2 lesions, similar results for both the PTP and OLE were observed, with mean values of 0 at the end of the first cycle for both ocrelizumab randomization groups, vs 1.4 and 2.1 in the placebo and IFN β-1a randomization groups, respectively (Table 2). Of the 141 participants with at least one post-ocrelizumab–treatment MRI assessment, 23 (16.3%) had new or enlarging T2 lesions recorded during the assessed TFP or at the OLE baseline assessment, with the first observed 24 weeks after the last dose of ocrelizumab (Fig. 4). New or enlarging T2 lesions off-treatment were observed more often in the placebo and IFN β-1a randomization groups than in those who received ocrelizumab in cycle 1: 38% (11/29) of the placebo randomization group and 32% (7/22) of the IFN β-1a randomization group with off-treatment assessments had new or enlarging lesions, vs 9% (4/45) of the ocrelizumab 2000 mg randomization group and 2% (1/45) of the ocrelizumab 600 mg randomization group.

Very few patients were still fully B-cell depleted at the time that MRI activity re-emerged, but there was no observable association between re-emergence of MRI activity and B-cell repletion level at thresholds of 5, 10, 40, or 80 cells/µL.

Mean brain volumes in patients receiving ocrelizumab are shown as BPF in Supplementary Table 2. Annualized loss in brain volume from baseline to treatment week 96 was approximately 0.4–0.8% in both periods (PTP and assessed TFP), with large standard deviations of approximately 0.7–0.9% around each point estimate.

ARR by treatment cycle (Supplementary Fig. 7) was significantly reduced during the first cycle for those receiving ocrelizumab compared with placebo [3]. Mean ARR was numerically higher during the first two treatment cycles among those in the ocrelizumab 2000 mg randomization group compared with the ocrelizumab 600 mg randomization group, but broadly comparable to the ocrelizumab 600 mg randomization group in cycles 3 and 4, when the ocrelizumab 2000 mg randomization group received 1000 mg and 600 mg, respectively. The ARR declined by approximately 65% in both the placebo and IFN β-1a randomization groups in cycle 2, following the first treatment with ocrelizumab 600 mg, to become similar in cycle 3 and numerically lower in cycle 4 to the rates seen among those randomized to ocrelizumab 600 mg. The ARR did not increase in any randomization group during the assessed TFP. The ARR in the TFP appeared numerically higher in the ocrelizumab 2000 mg randomization group than among those who received ocrelizumab 600 mg for three or four cycles. The ARR remained low in the OLE, comparable to, or lower than, the rates seen in the PTP and TFPs in the ocrelizumab 600 mg group.

**Fig. 5:**
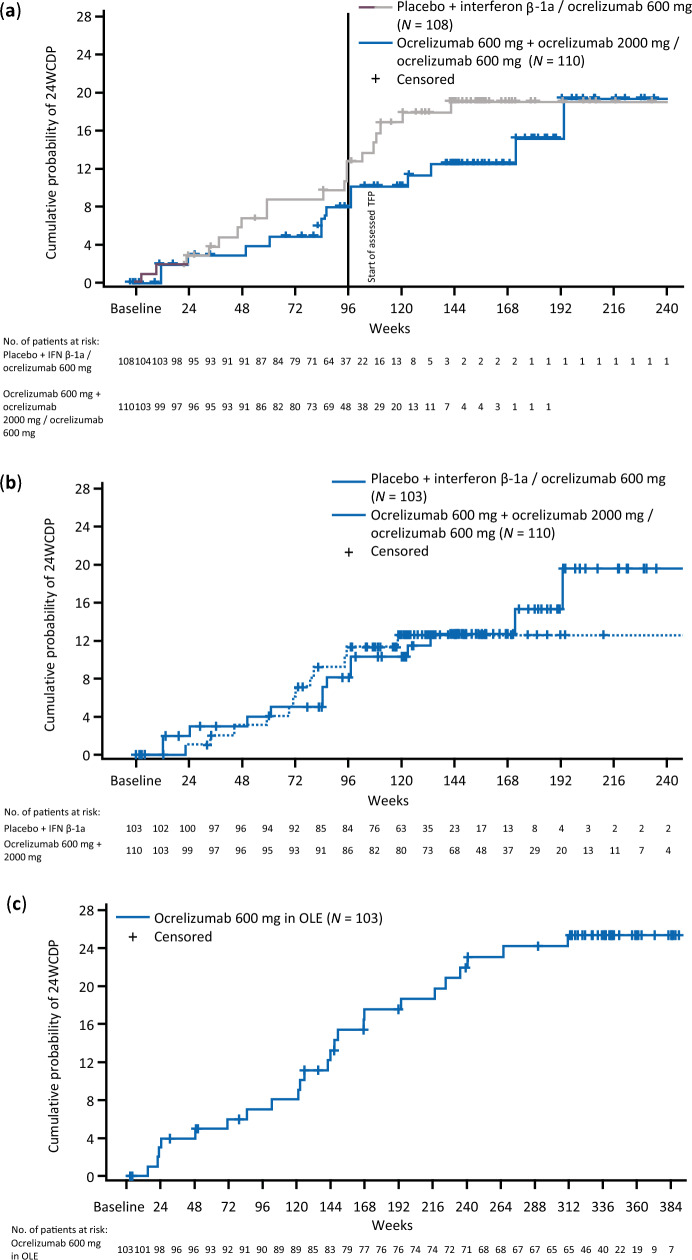
Time to onset of 24-week confirmed disability progression (**a**) the treatment and assessed TFP by randomization to ocrelizumab (600 or 2000 mg) or comparator (IFN β-1a or placebo) until end of assessed TFP. Last ocrelizumab treatment for all patients at week 72; **(b)** rebaselined to when patients started ocrelizumab (at randomization or at week 24); **(c)** from start of open-label extension (all patients on ocrelizumab). *24WCDP* confirmed disability progression of at least 24 weeks, *assessed TFP* assessed treatment-free period, *IFN* interferon, *PTP* primary treatment period

At week 96 in the PTP, 8.11% (2.72–13.51) of patients who started ocrelizumab in cycle 1 had 24WCDP, compared with 11.81% (5.53–18.10) of patients who received placebo or IFN β-1a in cycle 1; hazard ratio of 0.65 [0.32–1.30]; *p* = 0.2181, Fig. 5a. Among all participants entering the assessed TFP, the proportion of patients with 24WCDP at assessed TFP week 96 was 11.82% (4.66–18.98).

There was a trend toward shorter time to 24WCDP in the pooled comparator groups when assessed from the start of cycle 1 (hazard ratio 0.65 [0.32–1.30]; *p* = 0.2181; Fig. 5a). When assessing time to 24WCDP in the PTP and assessed TFP, from first ocrelizumab dose instead of the first cycle after randomization, it was found to be similar between pooled groups of those randomized to ocrelizumab or comparator (hazard ratio of ocrelizumab vs comparator, 0.92 [0.42–2.02]; *p* = 0.8332; Fig. 5b). In the OLE, the proportion of patients with 24WCDP at week 96 was 7.02% (2.00–12.03); the time to 24WCDP is shown in Fig. 5c.

**Fig. 6:**
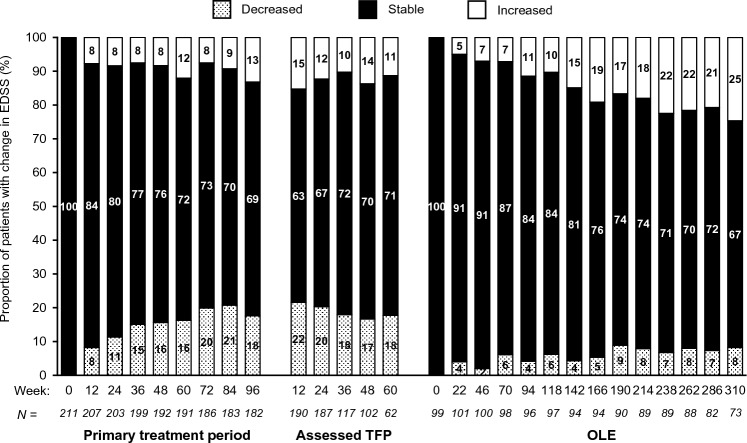
Categoric changes in EDSS for all patients by study visit in the PTP, assessed TFP, and OLE periods (rebaselined at start of OLE). PTP and assessed TFP data are relative to study week 0. Participants in the OLE were rebaselined at OLE week 0, with the baseline defined as the last evaluable EDSS assessment before the first OLE infusion. OLE assessments were performed 2 weeks before dosing visits. Changes were classified using CDP criteria of ±  ≥ 1 EDSS point for an EDSS < 5.5, and ±  0.5 points for EDSS ≥ 5.5. Only visits with data for > 50 subjects are shown for the assessed TFP and OLE. *Assessed TFP* assessed treatment-free period, *CDP* confirmed disability progression, *EDSS* Expanded Disability Status Scale, *OLE* open-label extension, *PTP* primary treatment period, *TFP* treatment-free period

The distribution of aggregated EDSS values among all participants was also broadly similar across visits for the PTP, assessed TFP, and the OLE, with the caveat of attrition bias with low participant numbers for later visits (Supplementary Fig. 8). Similarly, categoric changes in EDSS from study baseline (stable, decreased, or increased) using CDP criteria (± 1 EDSS point for baseline EDSS < 5.5, and ± 0.5 points for baseline EDSS ≥ 5.5) remained essentially stable across the PTP and assessed TFP. There appeared to be a trend toward higher proportions of increased scores at later visits in the OLE relative to OLE baseline (Fig. 6), though again with the caveat of attrition bias.

## Discussion

With a maximum treatment and study duration of ~ 9 and ~ 11 years, respectively, this report represents the longest available follow-up of ocrelizumab-treated patients with RRMS.

The long-term safety profile of ocrelizumab observed in the OLE of this phase 2 trial was in line with the favorable safety profile observed in the core part and prolonged treatment period of the trial, as well as those reported in the pivotal trials in RMS and PPMS [1, 2]; no new safety signals emerged. This study also established the median 71.9-week B-cell repletion timeframe with a threshold of 80 cells/µL. MRI activity, ARR, and CDP remained low over all study periods. Over longer periods of treatment interruption, there was no indication of rebound activity, and only a few participants had signs of disease reactivation. This supports an important advantage over other approved MS drugs, especially those that interfere with immune cell trafficking [9, 10]. The most common AEs reported in this phase 2 study were infusion-related reactions. The highest incidence of infusion-related reactions in both the PTP and OLE was observed with the first ocrelizumab infusion and declined over time, consistent with the observation in the phase 3 studies and their long-term extensions [11]. Over the overall observation period, we neither observed an increase in the incidence of any AEs nor any new emergent safety signals, including no cases of PML. Two cases of malignancies, one of which was present prestudy, were observed. This is in line with analyses of malignancy and female breast cancer rates in the overall clinical trial program for ocrelizumab and post-marketing data that do not suggest an increased or time-dependent exposure risk compared with matched reference MS populations or general populations [12, 13].

The higher rate of AEs and SAEs in the OLE compared with the TFP is not surprising, given the fact that patients did not receive treatment during the TFP. Certain types of AE, such as infusion-related reactions, cannot, by definition, occur in the TFP. The lower rate of AEs, SAEs, and serious infections observed in both the assessed TFP and OLE compared with the PTP may be due to lower assessment frequencies, underreporting, and OLE selection bias for participants with a favorable PTP response. Therefore, no definite conclusions on the relative frequency of AEs in the different periods can be drawn.

Laboratory data were as expected based on the pharmacologic mechanism of ocrelizumab, including depletion of total B cells and B-cell subsets. Slow declines in Ig concentrations across the OLE (observed yearly rates approximately − 2.4% over 7 years for IgG) were consistent with those reported in the long-term follow-up of the pivotal studies, where a reduction at a mean rate of 3 to 4%/year for serum IgG was observed [8]. Clinical trial data have shown a variable association between decreased levels of IgG and serious infections [14]; however, for most patients in this phase 2 study, Ig levels remained above the lower limit of normal, and overall T-cell populations remained stable throughout the study.

As previously reported [15], patients randomized to ocrelizumab from the start of the study showed a rapid suppression of new MRI activity within 4–8 weeks, which was maintained over the entire PTP. During the assessed TFP, return of MRI activity was observed in only a few patients, the earliest seen 24 weeks after the last ocrelizumab dose. As previously observed, B-cell repletion was associated with persistent reductions in the proportion of circulating memory B cells and plasmablasts/plasma cells compared with pre-ocrelizumab treatment levels, which might account for the long-lasting reductions in disease activity observed during the assessed TFP.

After re-initiation of ocrelizumab treatment in OLE, MRI activity was effectively controlled again. ARR was also rapidly suppressed among those randomized to ocrelizumab from study start [3], and was similarly suppressed in the comparator groups after switching to ocrelizumab. In the PTP, the ARR was numerically higher in the ocrelizumab 2000 mg randomization group and remained higher during the assessed TFP. This was driven by a few patients with a 2–3 × higher relapse rate, also associated with more MRI activity. During OLE, where all patients received 600 mg ocrelizumab, some patients randomized to the 2000 mg group also experienced a higher relapse rate, of note, not the same participants as those with higher ARR in the TFP. Comparison of baseline characteristics across all randomized groups did not reveal differences that would explain why this occurred. The overall relapse rate was very low in the assessed TFP and OLE, but the interpretation of these rates is limited, at least in part, by the same biases as discussed for the AEs (lower assessment frequencies with possible underreporting and selection of patients reacting favorably to ocrelizumab).

Overall CDP rates remained low across all study periods. After 36 weeks of the assessed TFP, a weak trend toward increase in absolute EDSS scores was noted. This stability may represent a residual ocrelizumab treatment effect during the period of B-cell repletion, further being supported by the observed trend toward earlier repletion of B cells in patients initially randomized to placebo/IFN β-1a, as the Kaplan–Meier analysis of 24WCDP by initial randomization group also indicates a trend toward a better outcome that persisted through the assessed TFP for those receiving ocrelizumab in cycle 1. Aggregated absolute EDSS scores remained broadly stable across the PTP, and although increasing EDSS scores might be expected during a treatment interruption, mean and median scores also remained broadly stable for the initial 36 weeks of the assessed TFP before a trend toward increase was noted (Fig. 6).

This study provides long-term data that further support the safety and early and sustained efficacy of ocrelizumab treatment, but comes with some important limitations. A phase 2 study has limited statistical power, and all analyses reported are exploratory. Only the first 24 weeks were strictly randomized and controlled. The heterogeneity of the OLE population, and the dosing complexity during the four PTP cycles together with the variable duration and different observation schedules of the assessed TFP and unassessed TFP complicate interpretation and comparison across the four treatment and untreated observation periods. Even with these inherent limitations, it is reassuring that no clinical disease reactivation was observed during the TFP, and only a few patients showed return of MRI activity on brain scans, with the earliest detected at week 24 after the last dose.

Given the relation to the overall observation period of more than 10 years, the failure to show a significant effect of ocrelizumab treatment delay by 6 months on disability outcomes is not surprising. In the OPERA and ORATORIO studies, a delay of ocrelizumab treatment by ≥ 2 years resulted in significantly worse long-term outcomes [1, 2]. This, together with the overall manageable AE profile seen in this study with a median cumulative ocrelizumab exposure of 1.8 years in the PTP and 6.5 years in the OLE period, further encourages the exploration of the benefits of early and continuous treatment.

With an overall median duration of the TFP of 92.6 weeks (IQR 60.8–131.0), this study also provided an opportunity to assess the effects of treatment interruption. The observed stability, even over prolonged periods without treatment, is reassuring from a safety perspective and might also inform the debate about the timing of SARS-CoV-2 vaccination, since there is evidence that humoral responses to vaccination improve with increasing interval between the last dose of B-cell–depleting therapy and vaccination [16, 17]. This study does not answer the question of whether the currently approved dosing regimen with ocrelizumab could be changed. Because of the inhomogeneous population, the attrition over 10 years, the low and variable frequency of assessments, and the relative low case numbers, the power of this study for detection of more subtle signs of disease reactivation or progression of disability was low. Findings from the pivotal studies, where higher exposure was associated with a decreased risk for future CDP [18], underline that our data should be interpreted with caution in the discussion about possible advantages and disadvantages of treatment interruption or less frequent dosing. Even if no apparent reactivation was observed for relapses, there may still be a higher risk of disease progression with lower ocrelizumab exposure. Further studies are needed to better define the optimal interval and dosing of ocrelizumab in the long-term trajectory of MS. Two currently ongoing randomized controlled trials are testing the value of a higher dose of ocrelizumab in RMS (NCT04544436) and PPMS (NCT04548999).

Overall, in this longest follow-up of ocrelizumab-treated patients with RRMS, no new safety signals, including no evidence of rebound following treatment interruption, were observed, supporting the positive benefit–risk balance of long-term ocrelizumab.

### Supplementary Information

Below is the link to the electronic supplementary material.

Supplementary file1 (DOCX 30 KB)

Supplementary file2 (DOCX 58 KB)

Supplementary file3 (DOCX 132 KB)

Supplementary file4 (DOCX 129 KB)

Supplementary file5 (DOCX 222 KB)

Supplementary file6 (DOCX 54 KB)

Supplementary file7 (DOCX 84 KB)

Supplementary file8 (DOCX 126 KB)

Supplementary file9 (DOCX 23 KB)

Supplementary file10 (PDF 16 KB)

Supplementary file11 (DOCX 137 KB)

Supplementary file12 (DOCX 140 KB)

## Data Availability

Qualified researchers may request access to individual patient-level data through the clinical study data request platform (https://vivli.org/). Further details on Roche’s criteria for eligible studies are available here: (https://vivli.org/members/ourmembers/). For further details on Roche’s Global Policy on the Sharing of Clinical Information and how to request access to related clinical study documents, see here: https://www.roche.com/research_and_development/who_we_are_how_we_work/clinical_trials/our_commitment_to_data_sharing.htm. Study WA21493 (NCT00676715) is listed on Vivli already and data can be requested for download. The data supporting the manuscript will be made available upon request after publication of the manuscript. A data dictionary will be shared, including the dataset specification of the study data tabulation model and annotated electronic case report forms (eCRFs) for the data up to the 2020 cut. A clinical study report including data up to 2015 is also available. The shared data will be de-identified participant data. Data will be made available on the secure environment on Vivli. Information about the sharing process, what can be shared with whom, etc., can be found under the Roche member section of the Vivli homepage (https://vivli.org/).
