# Supplementary material for: Ocrelizumab exposure in relapsing–remitting multiple sclerosis: 10-year analysis of the phase 2 randomized clinical trial and its extension
Source: J Neurol. 2023 Oct 31;271(2):642–57. doi: 10.1007/s00415-023-11943-4 (PMC10827899; doi:10.1007/s00415-023-11943-4)
Supplement: Supplementary file 1 — Supplementary file1 (DOCX 30 KB) [file 415_2023_11943_MOESM1_ESM.docx]

**Ocrelizumab exposure in relapsing–remitting multiple sclerosis: 10-year analysis of the phase 2 randomized clinical trial and its extension**

**Journal of Neurology**

**Authors: Ludwig Kappos, Anthony Traboulsee, David K.B. Li, Amit Bar-Or, Frederik Barkhof, Xavier Montalban, David Leppert, Anna Baldinotti, Hans-Martin Schneble, Harold Koendgen, Annette Sauter, Qing Wang, Stephen L. Hauser**

**Corresponding author:
Prof. Ludwig Kappos, MD
Research Center for Clinical Neuroimmunology and Neuroscience Basel (RC2NB)
Departments of Head, Spine and Neuromedicine, Clinical Research, Biomedicine and Clinical Research,
University Hospital Basel
University of Basel, Basel
Switzerland
Email: ludwig.kappos@usb.ch**

**Supplementary methods**

**Study design**

**The primary treatment period**

In the primary treatment period (PTP), comprising four 24-week treatment cycles, participants were randomized 1:1:1:1 to receive double-blind ocrelizumab () (2,000 mg or 600 mg), placebo, or interferon (IFN) β-1a (open label) for one cycle, then dose-blinded ocrelizumab 1,000 mg or 600 mg for the remaining three cycles.

**The treatment-free period**

Participants completing all treatment cycles, and those who discontinued treatment early, entered an assessed treatment-free period (TFP) with 12-weekly assessments.

The first 24 weeks of the assessed TFP assessed safety and B-cell repletion; any participant with persistent depletion by week 24 (repletion was defined as the number of B cells ≥ 80 cells/µL, or a return to baseline values, whichever was lower) entered a period of extended B-cell monitoring until repletion. After repletion, participants were monitored for an additional 24 weeks to complete the assessed TFP.

A minimum assessed TFP duration of 48 weeks, regardless of B-cell depletion, was required to enter the open-label extension (OLE). However, most entering the OLE had already completed the assessed TFP with repletion and initiated OLE dosing after a variable unassessed TFP interval (2 weeks to 2.6 years from end of assessed TFP to initiation of OLE), without prospective data capture (Supplementary Fig. 2).

**The open-label extension**

Participants completing both the PTP and at least 48 weeks of the assessed TFP (regardless of their B-cell repletion status) were eligible to enter the open-label extension (OLE), upon reconsenting. Participants received intravenous ocrelizumab 300 mg at OLE baseline and OLE week 2, followed by intravenous ocrelizumab 600 mg every 24 weeks thereafter. Those who were not eligible, or who declined to enter the OLE, completed the trial after B-cell repletion in the assessed TFP. The first patient entered the OLE on March 15, 2012 and the last on December 12, 2013. The OLE is ongoing at the time of writing.

**Procedures**

Brain MRI scans (proton density and T2-weighted images; T1-weighted images before and after gadolinium administration) were obtained in all participants at baseline and every 4 weeks through the first treatment cycle in the PTP, at the end of the treatment period (week 96), and at week 48 of the assessed TFP (study week 144) in the subset of participants randomized to receive ocrelizumab 2,000 mg or 600 mg. In the OLE, a baseline MRI was performed in all participants who did not have a final (week 144) MRI assessment in the assessed TFP, and in all available participants a further MRI at OLE week 96 was performed. MRI scans were assessed centrally for endpoint analysis. MRI scans taken up to and including OLE week 96 were assessed by the Multiple Sclerosis/MRI Research Group at the University of British Columbia, Canada. Starting at OLE week 120, MRIs were being read locally.

In addition to central assessment of MRI endpoints, all MRI scans were also reviewed by the site radiologist to identify any new relevant abnormalities not consistent with multiple sclerosis, particularly with respect to possible cases of progressive multifocal leukoencephalopathy.

Brain parenchymal fraction was determined using an automated in-house method, based on the multimodal segmentation of registered proton density and T2 image pairs [1].

Protocol-defined relapse was assessed by the treating investigator at each visit, and at unscheduled visits where required. It was defined as the occurrence of persistent (> 24 hours) new or worsening neurologic symptoms attributable to multiple sclerosis that followed at least 30 days of stable or improving neurologic state. These symptoms must have been accompanied by objective neurologic worsening consistent with an increase of at least half a step on the Expanded Disability Status Scale (EDSS), or two points on one of the appropriate Functional Systems Scores (FSS), or one point on two or more of the appropriate FSS.

Confirmed disability progression was defined as an EDSS increase of at least 1 point from baseline (0.5 points if baseline score was ≥ 5.5), confirmed for 12 or 24 weeks as determined by regular visits.

All EDSS and FSS evaluations were performed by a qualified examining investigator, trained and certified in administering FSS and EDSS [2] prior to study start, who had no access to other study or patient-related information.

B-cell subsets were measured using a panel developed by Q2Labs and included total CD19+ B cells, and CD19+ CD38lo CD27+ memory B cells.

**Supplementary results**

Two malignancies were observed in total, and both were classed as serious adverse events. The first was a 53-year-old woman (ocrelizumab 2,000 mg randomization group) who presented with a pre-existing leg lesion at study day 2 that was later identified as grade 2 squamous cell carcinoma following a skin excision biopsy on day 106. The patient was withdrawn from the study on day 171 and subsequently completed the assessed TFP. The patient was followed up until B-cell repletion was achieved (final study visit was on day 577). The second was a case of grade 3 breast carcinoma identified on study day 748 in a 43-year-old woman (ocrelizumab 2,000 mg randomization group) who had completed the PTP and was diagnosed shortly after entering the assessed TFP.

There were four deaths at time of data cut off. One death due to systemic inflammatory response syndrome of unknown cause occurred on study day 92 during the PTP phase in a 41-year-old woman (ocrelizumab 2,000 mg randomization group) [3]. Two deaths occurred in the assessed TFP in participants who had completed the PTP – one in a 32-year-old man (randomized to placebo) due to a fall on study day 968, and one in a 34-year-old man (ocrelizumab 600 mg randomization group) from unknown causes on study day 1,074. The fourth death (urosepsis) occurred during the OLE (study day 2,931) in a 49-year-old woman (ocrelizumab 2,000 mg randomization group).

**References**

1. Vavasour IM, Tam R, Li DKB et al (2019) A 24-month advanced magnetic resonance imaging study of multiple sclerosis patients treated with alemtuzumab. Mult Scler 25:811–818.

2. Neurostatus.net (2016) Kurtzke’s Functional Systems and the Expanded Disability Status Scale in ultiple Sclerosis. [www.neurostatus.net](http://www.neurostatus.net) Accessed 19 October 2021.

3. Kappos L, Li D, Calabresi PA et al (2011) Ocrelizumab in relapsing-remitting multiple sclerosis: a phase 2, randomized, placebo-controlled, multicentre trial. Lancet 378:1779–1787.
