# Supplementary material for: Ocrelizumab exposure in relapsing–remitting multiple sclerosis: 10-year analysis of the phase 2 randomized clinical trial and its extension
Source: J Neurol. 2023 Oct 31;271(2):642–57. doi: 10.1007/s00415-023-11943-4 (PMC10827899; doi:10.1007/s00415-023-11943-4)
Supplement: Supplementary file 2 — Supplementary file2 (DOCX 58 KB) [file 415_2023_11943_MOESM2_ESM.docx]

**Ocrelizumab exposure in relapsing–remitting multiple sclerosis: 10-year analysis of the phase 2 randomized clinical trial and its extension**

**Journal of Neurology**

**Authors: Ludwig Kappos, Anthony Traboulsee, David K.B. Li, Amit Bar-Or, Frederik Barkhof, Xavier Montalban, David Leppert, Anna Baldinotti, Hans-Martin Schneble, Harold Koendgen, Annette Sauter, Qing Wang, Stephen L. Hauser**

**Corresponding author:
Prof. Ludwig Kappos, MD
Research Center for Clinical Neuroimmunology and Neuroscience Basel (RC2NB)
Departments of Head, Spine and Neuromedicine, Clinical Research, Biomedicine and Clinical Research,
University Hospital Basel
University of Basel, Basel
Switzerland
Email: ludwig.kappos@usb.ch**


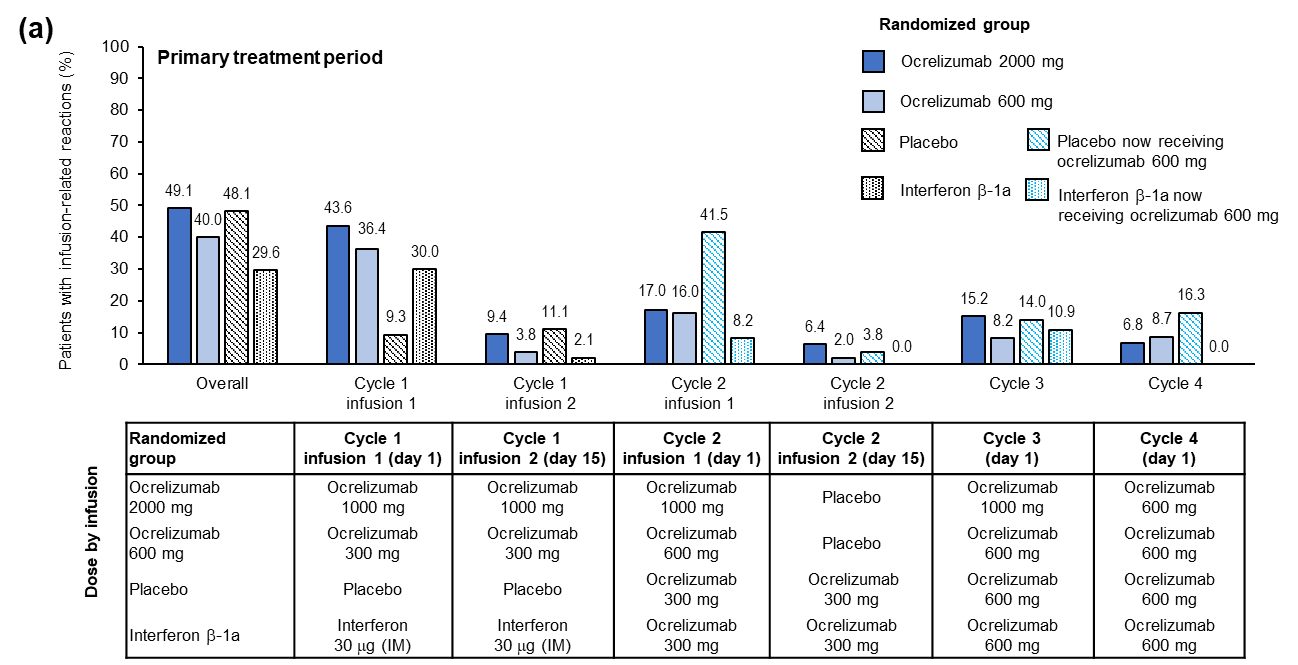


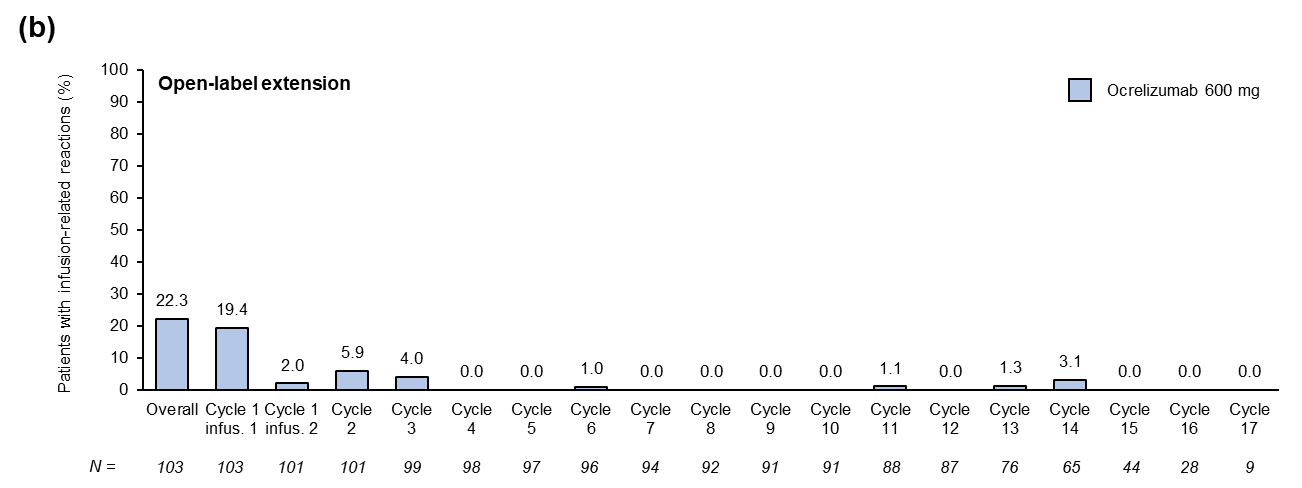


**Supplementary Fig. 3** Number (%) of patients with infusion-related reactions overall and by individual infusions given in (**a**) the PTP, and (**b**) the OLE period

Patients randomized to the IFN β-1a treatment arm received weekly IM dosing of IFN for the first 24-week treatment cycle before switching to ocrelizumab 600 mg infusions for cycles 2–4; reactions in this group for cycle 1 refer to IM administration of IFN β-1a on weeks 1 and 2

*IFN* interferon, *IM* intramuscular, *infus.* infusion, *OLE* open-label extension, *PTP* primary treatment period
