# Supplementary material for: Ocrelizumab exposure in relapsing–remitting multiple sclerosis: 10-year analysis of the phase 2 randomized clinical trial and its extension
Source: J Neurol. 2023 Oct 31;271(2):642–57. doi: 10.1007/s00415-023-11943-4 (PMC10827899; doi:10.1007/s00415-023-11943-4)
Supplement: Supplementary file 3 — Supplementary file3 (DOCX 132 KB) [file 415_2023_11943_MOESM3_ESM.docx]

**Ocrelizumab exposure in relapsing–remitting multiple sclerosis: 10-year analysis of the phase 2 randomized clinical trial and its extension**

**Journal of Neurology**

**Authors: Ludwig Kappos, Anthony Traboulsee, David K.B. Li, Amit Bar-Or, Frederik Barkhof, Xavier Montalban, David Leppert, Anna Baldinotti, Hans-Martin Schneble, Harold Koendgen, Annette Sauter, Qing Wang, Stephen L. Hauser**

**Corresponding author:
Prof. Ludwig Kappos, MD
Research Center for Clinical Neuroimmunology and Neuroscience Basel (RC2NB)
Departments of Head, Spine and Neuromedicine, Clinical Research, Biomedicine and Clinical Research,
University Hospital Basel
University of Basel, Basel
Switzerland
Email: ludwig.kappos@usb.ch**


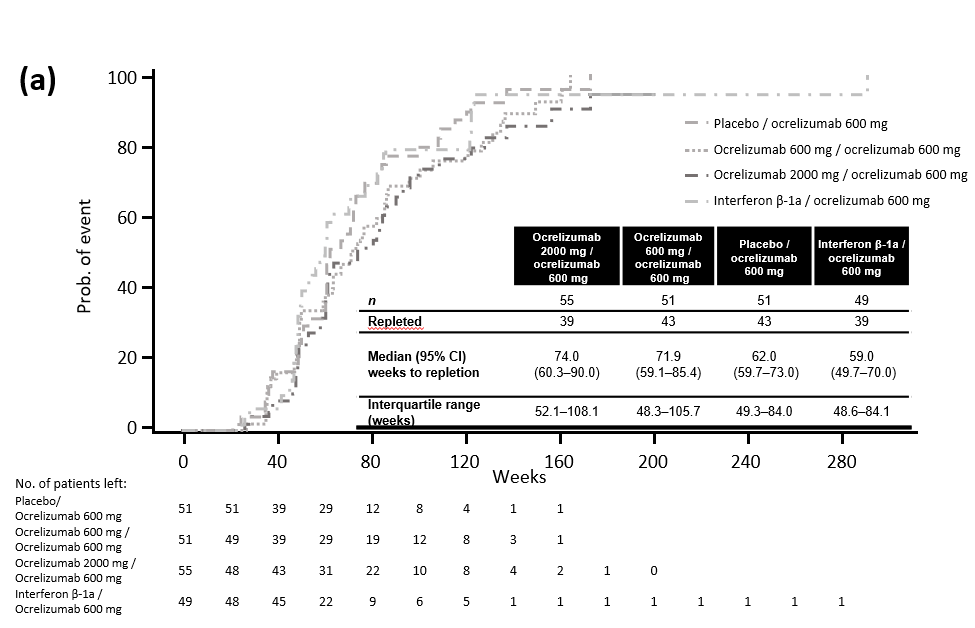


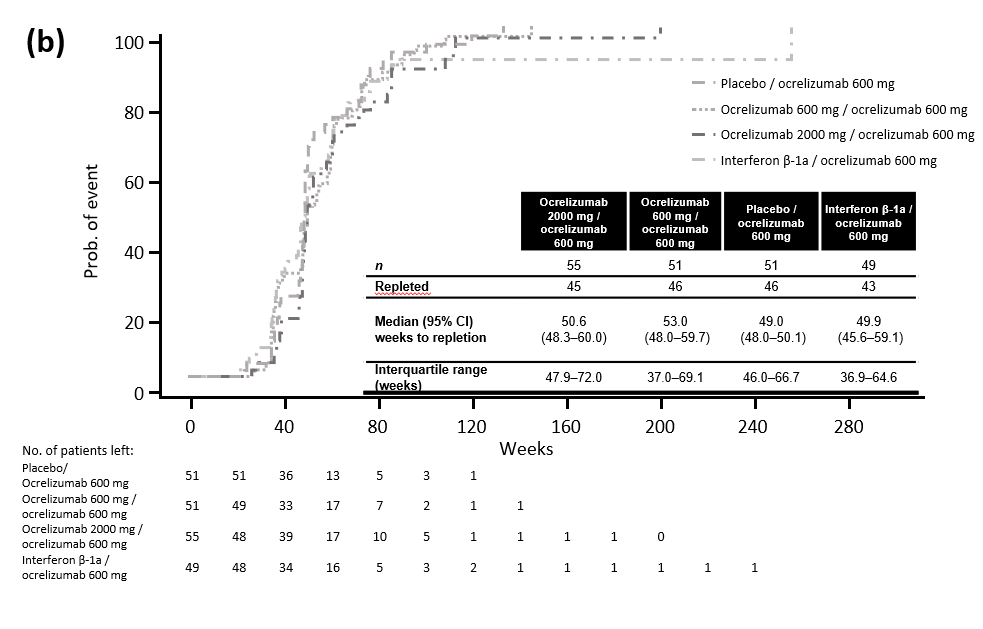


**Supplementary Fig. 4** Cumulative probability of B-cell (CD19+) repletion in the assessed treatment-free period by initial randomization group using repletion thresholds of (**a**) 80 cells/µL or (**b**) 40 cells/µL

Repletion was defined as a return to the pretreatment baseline or to above the LLN, whichever was lower. The LLN is based on healthy donor reference ranges that can vary by laboratory, thus two common LLN thresholds are shown

Medians are Kaplan–Meier estimates. Ranges are based on observed values. Weeks are relative to the date of the last dose of ocrelizumab

*CI* confidence interval, *IFN* interferon, *IQR* interquartile range, *LLN* lower limit of normal, *prob.* probability
