# Supplementary material for: Ocrelizumab exposure in relapsing–remitting multiple sclerosis: 10-year analysis of the phase 2 randomized clinical trial and its extension
Source: J Neurol. 2023 Oct 31;271(2):642–57. doi: 10.1007/s00415-023-11943-4 (PMC10827899; doi:10.1007/s00415-023-11943-4)
Supplement: Supplementary file 4 — Supplementary file4 (DOCX 129 KB) [file 415_2023_11943_MOESM4_ESM.docx]

**Ocrelizumab exposure in relapsing–remitting multiple sclerosis: 10-year analysis of the phase 2 randomized clinical trial and its extension**

**Journal of Neurology**

**Authors: Ludwig Kappos, Anthony Traboulsee, David K.B. Li, Amit Bar-Or, Frederik Barkhof, Xavier Montalban, David Leppert, Anna Baldinotti, Hans-Martin Schneble, Harold Koendgen, Annette Sauter, Qing Wang, Stephen L. Hauser**

**Corresponding author:
Prof. Ludwig Kappos, MD
Research Center for Clinical Neuroimmunology and Neuroscience Basel (RC2NB)
Departments of Head, Spine and Neuromedicine, Clinical Research, Biomedicine and Clinical Research,
University Hospital Basel
University of Basel, Basel
Switzerland
Email: ludwig.kappos@usb.ch**

**
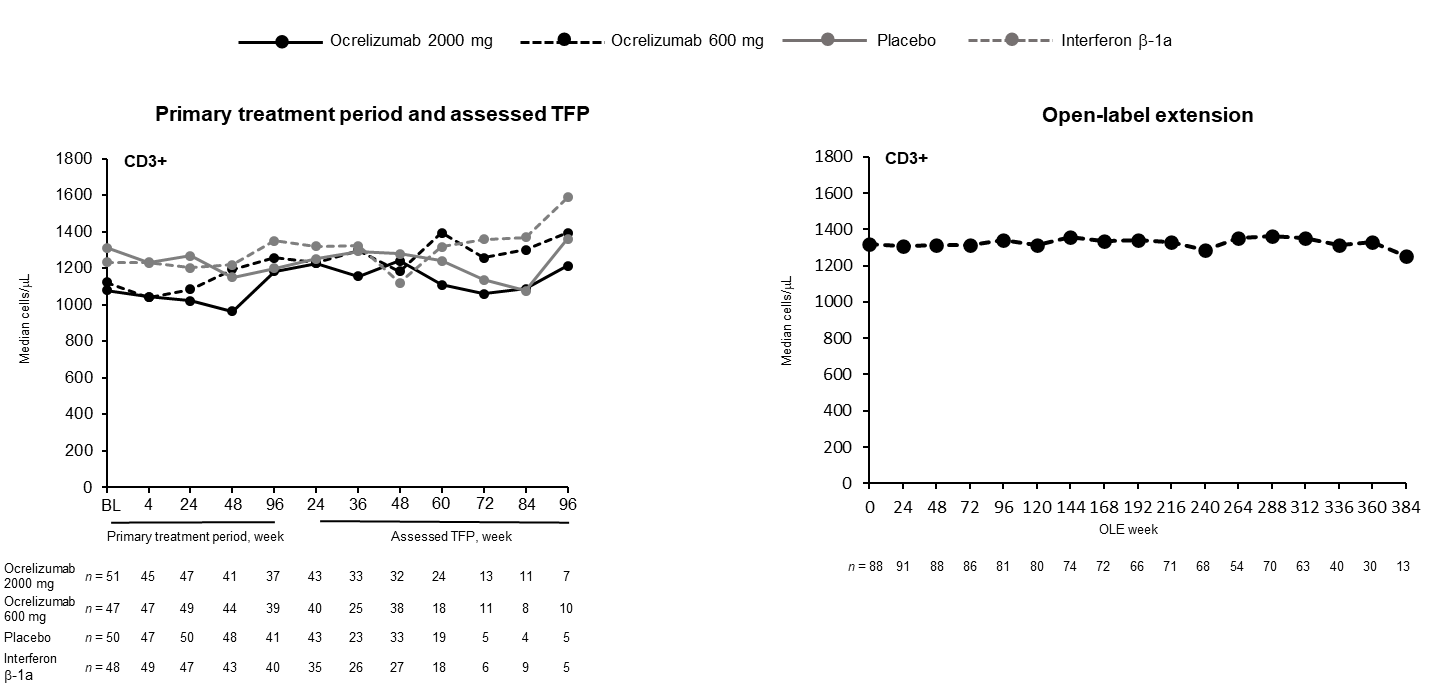
**

**
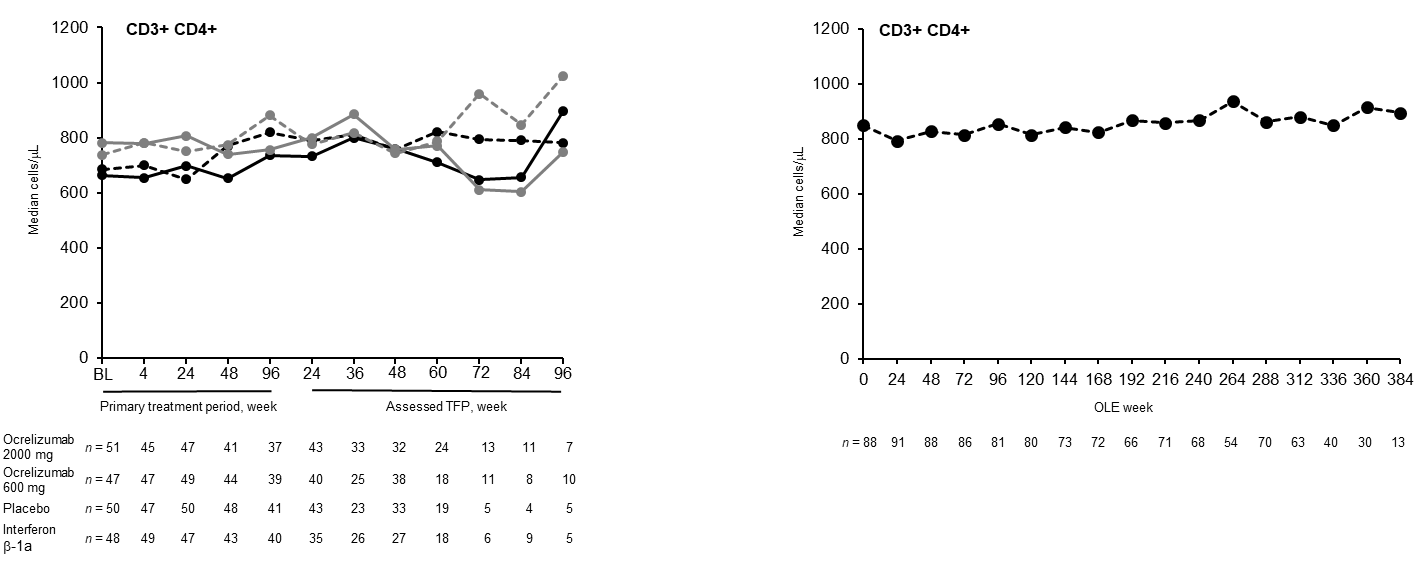
**

**
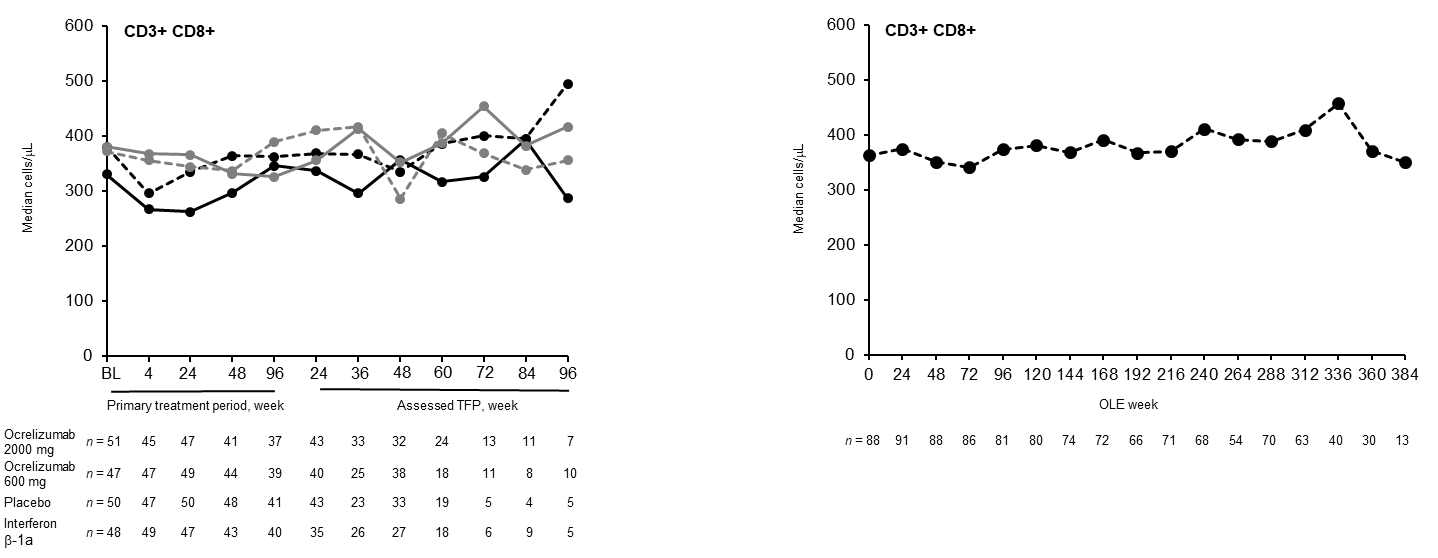
**

**Supplementary Fig. 5** Median CD3+ T-cell, CD3+ CD4+ T-cell, and CD3+ CD8+ T-cell counts during the PTP, assessed TFP, and OLE

Data for the PTP and assessed TFP are shown by initial randomization group

*Assessed TFP* assessed treatment-free period, *BL* baseline, *IFN* interferon, *OLE* open-label extension, *PBO* placebo, *PTP* primary treatment period
