# Supplementary material for: Ocrelizumab exposure in relapsing–remitting multiple sclerosis: 10-year analysis of the phase 2 randomized clinical trial and its extension
Source: J Neurol. 2023 Oct 31;271(2):642–57. doi: 10.1007/s00415-023-11943-4 (PMC10827899; doi:10.1007/s00415-023-11943-4)
Supplement: Supplementary file 5 — Supplementary file5 (DOCX 222 KB) [file 415_2023_11943_MOESM5_ESM.docx]

**Ocrelizumab exposure in relapsing–remitting multiple sclerosis: 10-year analysis of the phase 2 randomized clinical trial and its extension**

**Journal of Neurology**

**Authors: Ludwig Kappos, Anthony Traboulsee, David K.B. Li, Amit Bar-Or, Frederik Barkhof, Xavier Montalban, David Leppert, Anna Baldinotti, Hans-Martin Schneble, Harold Koendgen, Annette Sauter, Qing Wang, Stephen L. Hauser**

**Corresponding author:
Prof. Ludwig Kappos, MD
Research Center for Clinical Neuroimmunology and Neuroscience Basel (RC2NB)
Departments of Head, Spine and Neuromedicine, Clinical Research, Biomedicine and Clinical Research,
University Hospital Basel
University of Basel, Basel
Switzerland
Email: ludwig.kappos@usb.ch**


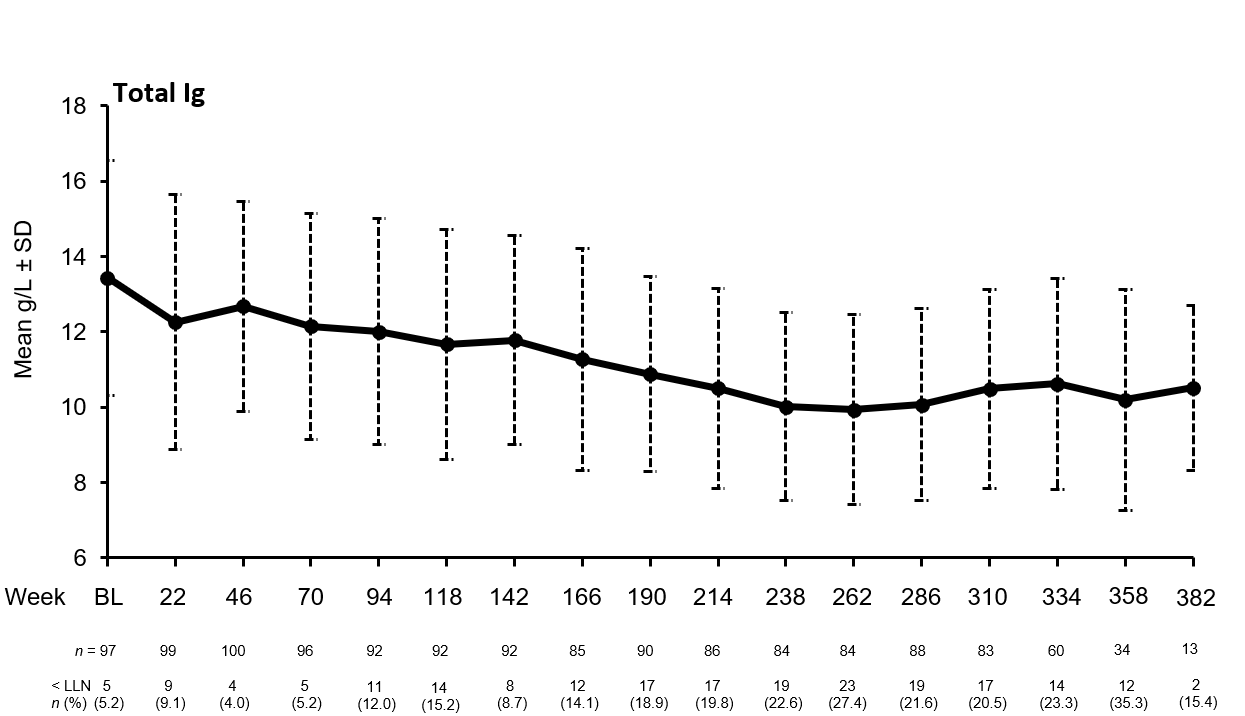


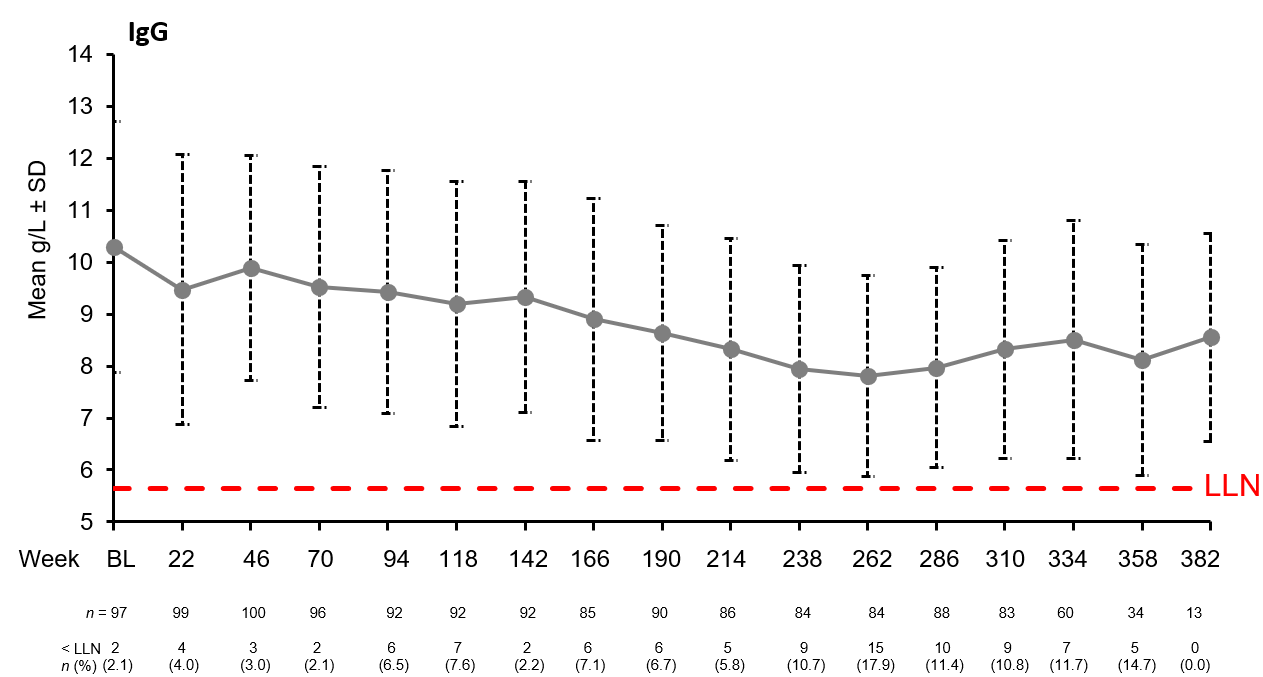


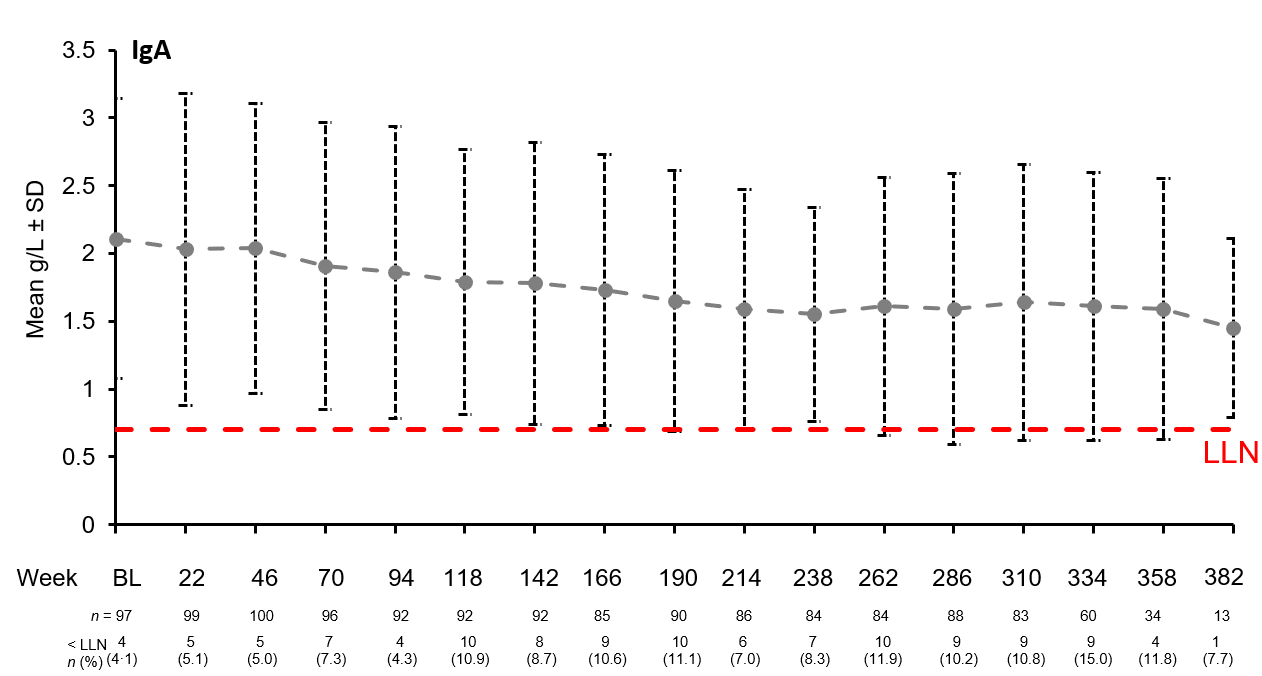


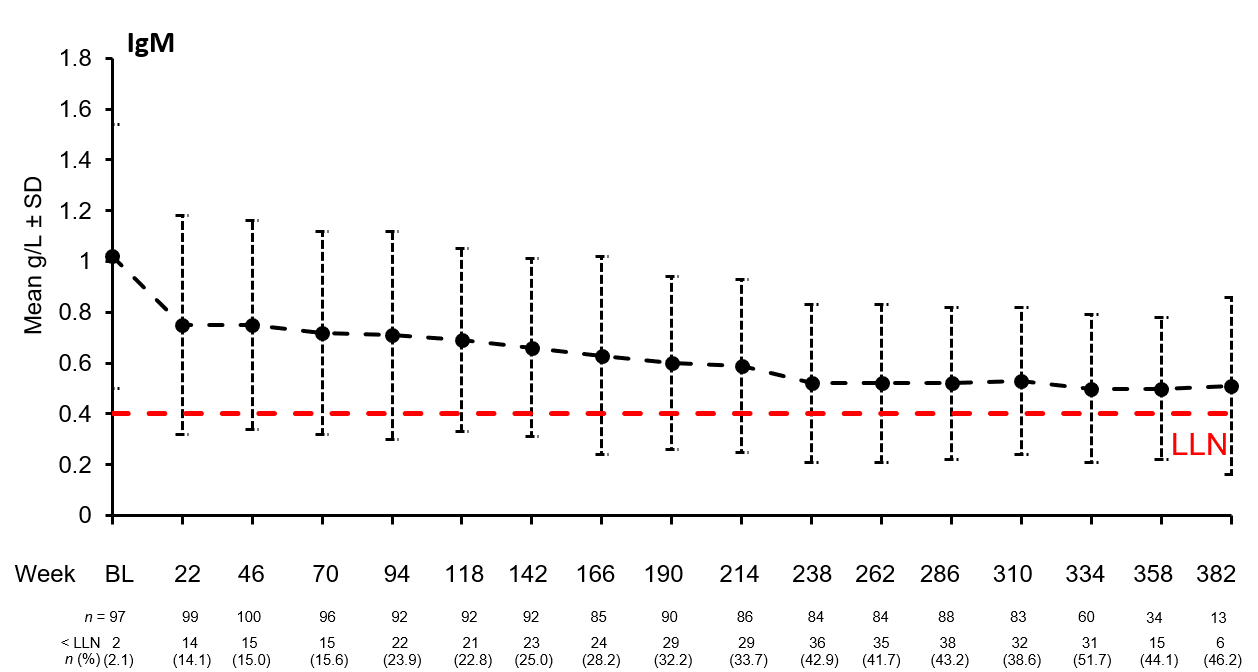


**Supplementary Fig. 6** Mean (SD) absolute values of immunoglobulin (Ig) fractions over time in the OLE period of 600 mg ocrelizumab every 24 weeks
 *BL* baseline, *Ig* immunoglobulin, *LLN* lower limit of normal, *OLE* open-label extension
