# Supplementary material for: Ocrelizumab exposure in relapsing–remitting multiple sclerosis: 10-year analysis of the phase 2 randomized clinical trial and its extension
Source: J Neurol. 2023 Oct 31;271(2):642–57. doi: 10.1007/s00415-023-11943-4 (PMC10827899; doi:10.1007/s00415-023-11943-4)
Supplement: Supplementary file 6 — Supplementary file6 (DOCX 54 KB) [file 415_2023_11943_MOESM6_ESM.docx]

**Ocrelizumab exposure in relapsing–remitting multiple sclerosis: 10-year analysis of the phase 2 randomized clinical trial and its extension**

**Journal of Neurology**

**Authors: Ludwig Kappos, Anthony Traboulsee, David K.B. Li, Amit Bar-Or, Frederik Barkhof, Xavier Montalban, David Leppert, Anna Baldinotti, Hans-Martin Schneble, Harold Koendgen, Annette Sauter, Qing Wang, Stephen L. Hauser**

**Corresponding author:
Prof. Ludwig Kappos, MD
Research Center for Clinical Neuroimmunology and Neuroscience Basel (RC2NB)
Departments of Head, Spine and Neuromedicine, Clinical Research, Biomedicine and Clinical Research,
University Hospital Basel
University of Basel, Basel
Switzerland
Email: ludwig.kappos@usb.ch**

**
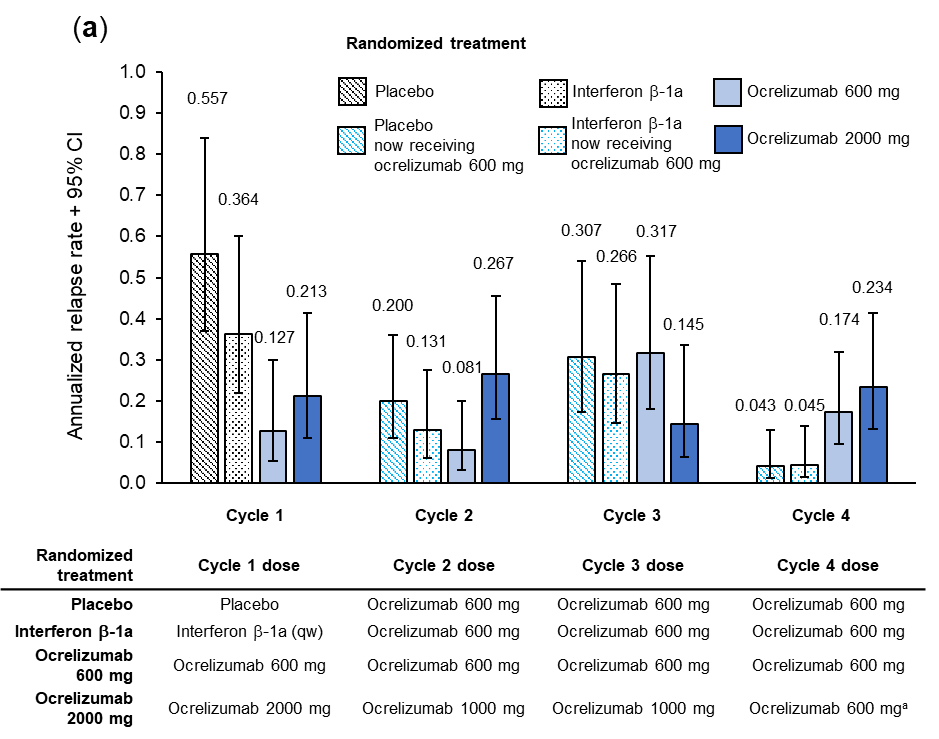
**


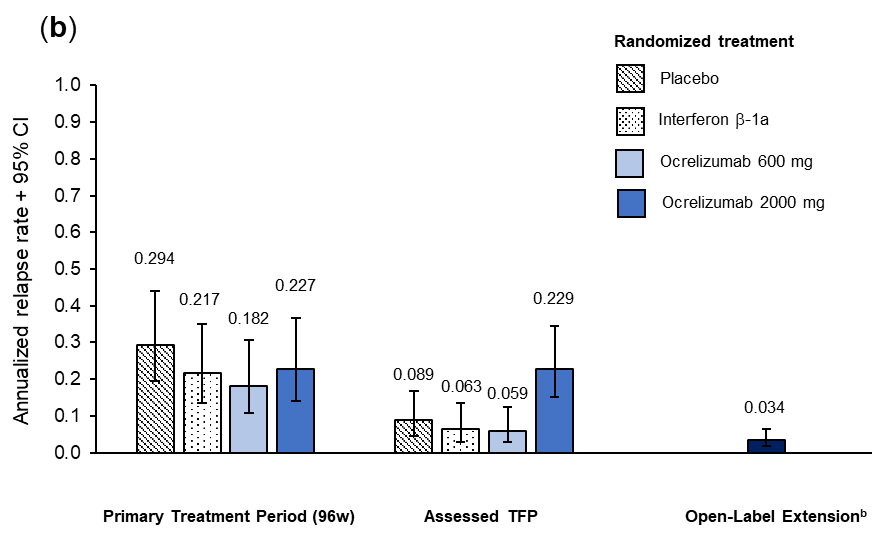


**Supplementary Fig. 7** **Annualized relapse rates** (Poisson regression adjusted for geographic region) with 95% CIs: (**a**) for each treatment cycle in the primary treatment period, and (**b**) by overall study period

^a^Four patients received 1000 mg

^b^Thirty relapses among 103 patients receiving ocrelizumab 600 mg every 24 weeks followed for 624 patient years in total
 *Assessed TFP* assessed treatment-free period, *CI* confidence interval, *qw* once weekly
