# Supplementary material for: Ocrelizumab exposure in relapsing–remitting multiple sclerosis: 10-year analysis of the phase 2 randomized clinical trial and its extension
Source: J Neurol. 2023 Oct 31;271(2):642–57. doi: 10.1007/s00415-023-11943-4 (PMC10827899; doi:10.1007/s00415-023-11943-4)
Supplement: Supplementary file 8 — Supplementary file8 (DOCX 126 KB) [file 415_2023_11943_MOESM8_ESM.docx]

**Ocrelizumab exposure in relapsing–remitting multiple sclerosis: 10-year analysis of the phase 2 randomized clinical trial and its extension**

**Journal of Neurology**

**Authors: Ludwig Kappos, Anthony Traboulsee, David K.B. Li, Amit Bar-Or, Frederik Barkhof, Xavier Montalban, David Leppert, Anna Baldinotti, Hans-Martin Schneble, Harold Koendgen, Annette Sauter, Qing Wang, Stephen L. Hauser**

**Corresponding author:
Prof. Ludwig Kappos, MD
Research Center for Clinical Neuroimmunology and Neuroscience Basel (RC2NB)
Departments of Head, Spine and Neuromedicine, Clinical Research, Biomedicine and Clinical Research,
University Hospital Basel
University of Basel, Basel
Switzerland
Email: ludwig.kappos@usb.ch**

**
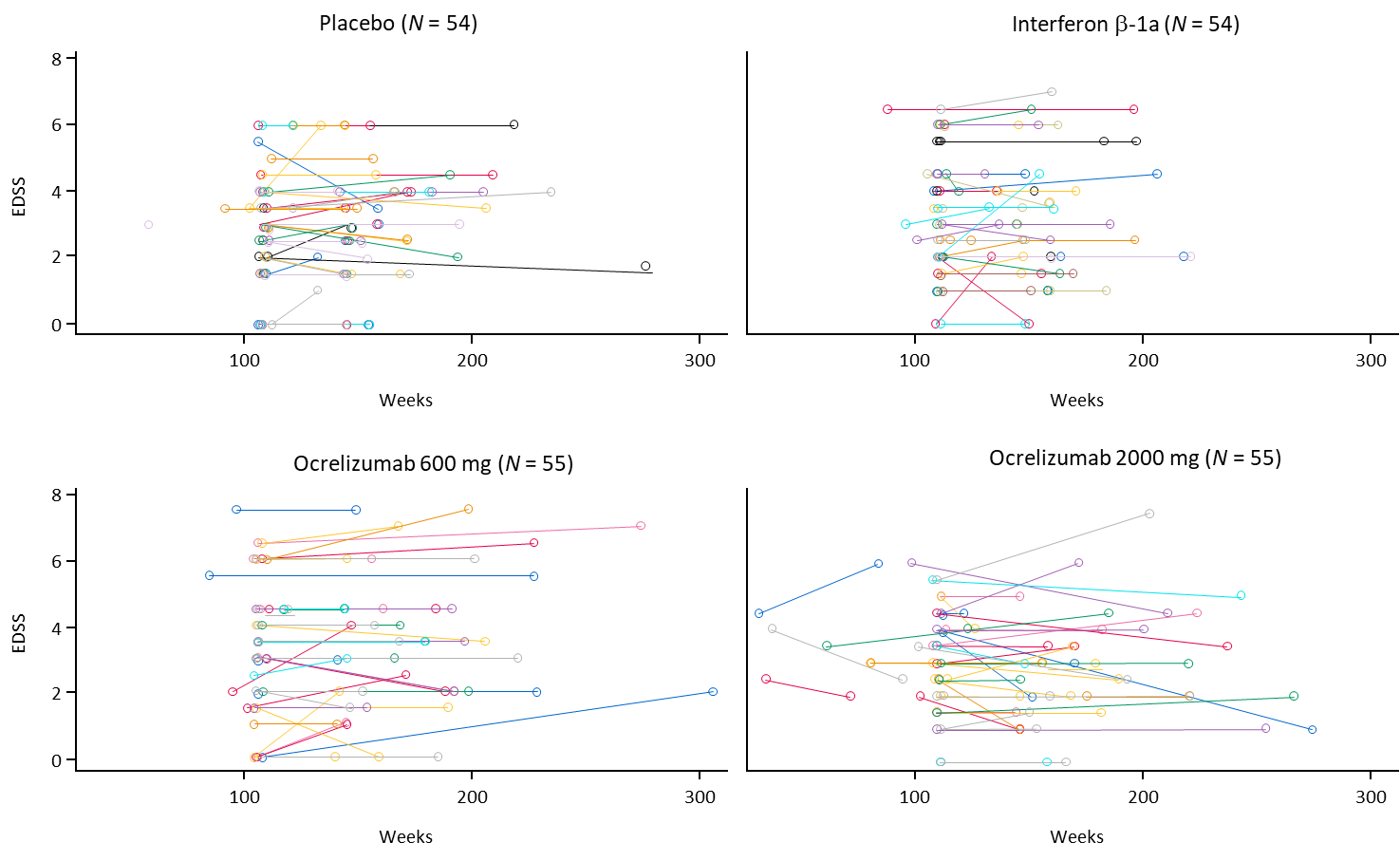
**

**Supplementary Fig. 9** EDSS during the treatment-free period by patient (first and last EDSS value during the treatment-free period, including OLE baseline)

*EDSS* Expanded Disability Status Scale, *OLE* open-label extension, *TFP* treatment-free period
