# Supplementary material for: Ocrelizumab exposure in relapsing–remitting multiple sclerosis: 10-year analysis of the phase 2 randomized clinical trial and its extension
Source: J Neurol. 2023 Oct 31;271(2):642–57. doi: 10.1007/s00415-023-11943-4 (PMC10827899; doi:10.1007/s00415-023-11943-4)
Supplement: Supplementary file 9 — Supplementary file9 (DOCX 23 KB) [file 415_2023_11943_MOESM9_ESM.docx]

**Ocrelizumab exposure in relapsing–remitting multiple sclerosis: 10-year analysis of the phase 2 randomized clinical trial and its extension**

**Journal of Neurology**

**Authors: Ludwig Kappos, Anthony Traboulsee, David K.B. Li, Amit Bar-Or, Frederik Barkhof, Xavier Montalban, David Leppert, Anna Baldinotti, Hans-Martin Schneble, Harold Koendgen, Annette Sauter, Qing Wang, Stephen L. Hauser**

**Corresponding author:
Prof. Ludwig Kappos, MD
Research Center for Clinical Neuroimmunology and Neuroscience Basel (RC2NB)
Departments of Head, Spine and Neuromedicine, Clinical Research, Biomedicine and Clinical Research,
University Hospital Basel
University of Basel, Basel
Switzerland
Email: ludwig.kappos@usb.ch**

**Supplementary Table 1** Baseline characteristics of patients at the start of the PTP, of patients who did or did not subsequently enter the OLE, and of those who are still ongoing in the OLE

|  | **Primary treatment period** | | | | **Open-label population at randomization** | **Open-label population at entry into OLE** | **Patients still ongoing in OLE (*n* = 86)** | **Patients who did not enter the OLE (*n* = 115)** |
| --- | --- | --- | --- | --- | --- | --- | --- | --- |
|  | Ocrelizumab 2000 mg  (*n* = 55) | Ocrelizumab  600 mg  (*n* = 55) | Placebo  (*n* = 54) | Interferon  β-1a  (*n* = 54) | Ocrelizumab 600 mg  (*n* = 103) | |  |  |
| Age, median (range), y | 39.0 (21–56) | 35.0 (19–53) | 38.5 (22–54) | 38.0 (22–55) | 39.0 (21–54) | 43.0 (24–59) | 39.0 (21–54) | 38.0 (19–56) |
| Age group, *n* (%)  ≤ 45 y  > 45 to 65 y | 44 (80.0) 11 (20.0) | 48 (87.3) 7 (12.7) | 43 (79.6) 11 (20.4) | 38 (70.4) 16 (29.6) | 80 (77.7) 23 (22.3) | 66 (64.1) 37 (35.9) | 66 (76.7) 20 (23.3) | 93 (80.9) 22 (19.1) |
| Female sex, *n* (%) | 38 (69.1) | 35 (63.6) | 36 (66.7) | 32 (59.3) | 64 (62.1) | | 54 (62.8) | 77 (67.0) |
| Race, *n* (%)  White Black/African American Asian American Indian/Alaska native Other | 53 (96.4) 2 (3.6) 0 0 0 | 51 (92.7) 3 (5.5) 0 1 (1.8) 0 | 52 (96.3) 0 1 (1.9) 0 1 (1.9) | 53 (98.1) 1 (1.9) 0 0 0 | 98 (95.1) 3 (2.9) 1 (1.0) 0 1 (1.0) | | 83 (96.5) 1 (1.2) 1 (1.2) 0 1 (1.2) | 111 (96.5) 3 (2.6) 0 1 (0.9) 0 |
| Weight, median  (range), kg | 70.4 (40.0–116.0) | 70.0 (43.2–133.6) | 74.4 (45.0–139.0) | 73.0 (43.0–127.5) | 75.0 (40.0–133.6) | 75.0 (41.0–157.5) | 75.1  (40.0–133.6) | 70.0  (40.2–139.0) |
| Body mass index, median (range), kg/m^2^ | 24.4  (14.4–43.7) | 24.5  (16.3–52.8) | 24.4  (17.6–42.3) | 24.1  (16.7–51.1) | 24.8 (15.8–52.8) | 24.5  (16.8–62.3) | 24.6  (15.8–52.8) | 24.1  (14.4–51.1) |
| Region, *n* (%)  US  Rest of world | 11 (20.0) 44 (80.0) | 11 (20.0) 44 (80.0) | 11 (20.4) 43 (79.6) | 14 (25.9) 40 (74.1) | 14 (13.6) 89 (86.4) | | 11 (12.8)  75 (87.2) | 33 (28.7) 82 (71.3) |
| Subregion  Eastern-central Europe/Asia  North America  Western Europe | 30 (54.5) 17 (30.9)  8 (14.5) | 30 (54.5) 16 (29.1) 9 (16.4) | 30 (55.6) 16 (29.6) 8 (14.8) | 30 (55.6) 16 (29.6) 8 (14.8) | 62 (60.2) 23 (22.3) 18 (17.5) | | 51 (59.3) 19 (22.1)  16 (18.6) | 58 (50.4) 42 (36.5) 15 (13.0) |
| Duration since MS symptom onset, median (range), y | 7.7  (0.3–28.0) | 6.5  (0.5–20.5) | 4.8  (0.6–26.2) | 5.4  (0.8–35.2) | 6.7  (0.3–35.2) | 10.7  (3.7–38.6) | 6.6  (0.3–35.2) | 5.1  (0.5–26.2) |
| Duration since MS diagnosis, median (range), y | 4.4  (0.1–19.2) | 3.6  (0.1–16.5) | 2.7  (0.1–19.2) | 3.3  (0.1–20.2) | 3.52  (0.1–20.2) | 7.6  (3.4–23.7) | 3.5  (0.1–20.2) | 2.9  (0.1–19.2) |
| EDSS, median (range) | 3.5 (1.0–6.0) | 3.5 (1.0–6.0) | 3.0 (1.0–6.0) | 3.0 (1.0–6.0) | 3.5 (1.0–6.0) | 3.5 (0.0–7.5) | 3.5 (1.0–6.0) | 3.0 (1.0–6.0) |
| EDSS category, *n* (%)  ≤ 2.5  > 2.5 | 19 (34.5) 36 (65.5) | 19 (34.5) 36 (65.5) | 24 (44.4) 30 (55.6) | 26 (48.1) 28 (51.9) | 40 (38.8) 63 (61.2) | 43 (41.7) 60 (58.3) | 34 (39.5) 52 (60.5) | 48 (41.7) 67 (58.3) |
| Number of Gd-enhancing T1 lesions, *n/N* (%)  0  1–2  3–4  > 4 | 29/53 (54.7) 16/53 (30.2) 2/53 (3.8) 6/53 (11.3) | 25/51 (49.0) 12/51 (23.5) 7/51 (13.7) 7/51 (13.7) | 26/47 (55.3) 13/47 (27.7) 4/47 (8.5) 4/47 (8.5) | 33/50 (66.0) 9/50 (18.0) 1/50 (2.0) 7/50 (14.0) | 55/93 (59.1) 28/93 (30.1) 4/93 (4.3) 6/93 (6.5) | 95/98 (96.9) 2/98 (2.0) 0 1/98 (1.0) | 44/78 (56.4) 24/78 (30.8) 4/78 (5.1) 6/78 (7.7) | 58/108 (53.7) 22/108 (20.4) 10/108 (9.3) 18/108 (16.7) |
| Volume of T2 lesions, median (range), cm^3^ | 7.1  (0.2–59.4) | 6.7  (0.0–93.8) | 4.8  (0.0–39.9) | 8.3  (0.0–102.9) | 8.4  (0.0–102.9) | NA | 8.6  (0.0–102.9) | 6.2  (0.0–93.8) |

*EDSS* Expanded Disability Status Scale, *Gd* gadolinium, *MS* multiple sclerosis, *NA* not applicable, *OLE* open-label extension, *PTP* primary treatment period, *y* year
