# Supplementary material for: Ocrelizumab exposure in relapsing–remitting multiple sclerosis: 10-year analysis of the phase 2 randomized clinical trial and its extension
Source: J Neurol. 2023 Oct 31;271(2):642–57. doi: 10.1007/s00415-023-11943-4 (PMC10827899; doi:10.1007/s00415-023-11943-4)
Supplement: Supplementary file 10 — Supplementary file10 (PDF 16 KB) [file 415_2023_11943_MOESM10_ESM.docx]

**Ocrelizumab exposure in relapsing–remitting multiple sclerosis: 10-year analysis of the phase 2 randomized clinical trial and its extension**

**Journal of Neurology**

**Authors: Ludwig Kappos, Anthony Traboulsee, David K.B. Li, Amit Bar-Or, Frederik Barkhof, Xavier Montalban, David Leppert, Anna Baldinotti, Hans-Martin Schneble, Harold Koendgen, Annette Sauter, Qing Wang, Stephen L. Hauser**

**Corresponding author:
Prof. Ludwig Kappos, MD
Research Center for Clinical Neuroimmunology and Neuroscience Basel (RC2NB)
Departments of Head, Spine and Neuromedicine, Clinical Research, Biomedicine and Clinical Research,
University Hospital Basel
University of Basel, Basel
Switzerland
Email: ludwig.kappos@usb.ch**

**Supplementary Table 2** Brain parenchymal fraction by visit among those receiving ocrelizumab

| ***Mean BPF (SD) [n]*** | | |
| --- | --- | --- |
|  | **Ocrelizumab 2000 mg** | **Ocrelizumab 600 mg** |
| PTP baseline | 0.76 (0.06) [49] | 0.76 (0.06) [49] |
| PTP week 12 | 0.75 (0.06) [42] | 0.76 (0.06) [45] |
| PTP week 96 | 0.74 (0.06) [41] | 0.74 (0.06) [42] |
| OLE baseline | 0.73 (0.06) [88] | |
| OLE week 96 | 0.72 (0.06) [81] | |
| ***% Change in mean BPF (SD) [n]*** | | |
|  | **Ocrelizumab 2000 mg** | **Ocrelizumab 600 mg** |
| PTP baseline to PTP week 12 | –0.02 (1.08) [38] | –0.09 (1.31) [41] |
| PTP week 12 to PTP week 96 | –1.43 (1.69) [33] | –1.31 (1.24) [39] |
| PTP baseline to PTP week 96 | –1.43 (1.63) [36] | –1.55 (1.58) [37] |
| OLE baseline to OLE week 96 | –0.97 (1.55) [75] | |

| ***% Annualized change in mean BPF (SD) [n]*** | | |
| --- | --- | --- |
|  | **Ocrelizumab 2000 mg** | **Ocrelizumab 600 mg** |
| PTP baseline to PTP week 12 | –0.10 (4.12) [38] | –0.35 (4.68) [41] |
| PTP week 12 to PTP week 96 | – 0.89 (1.05) [33] | –0.81 (0.77) [39] |
| PTP baseline to PTP week 96 | –0.77 (0.88) [36] | –0.84 (0.85) [37] |
| OLE baseline to OLE week 96 | –0.42 (0.71) [75] | |

OLE baseline is the last evaluable MRI measure between the week 144 visit and the first infusion in the OLE

*BPF* brain parenchymal fraction, *OLE* open-label extension, *PTP* primary treatment period
