# Supplementary material for: Ocrelizumab exposure in relapsing–remitting multiple sclerosis: 10-year analysis of the phase 2 randomized clinical trial and its extension
Source: J Neurol. 2023 Oct 31;271(2):642–57. doi: 10.1007/s00415-023-11943-4 (PMC10827899; doi:10.1007/s00415-023-11943-4)
Supplement: Supplementary file 11 — Supplementary file11 (DOCX 137 KB) [file 415_2023_11943_MOESM11_ESM.docx]

**Ocrelizumab exposure in relapsing–remitting multiple sclerosis: 10-year analysis of the phase 2 randomized clinical trial and its extension**

**Journal of Neurology**

**Authors: Ludwig Kappos, Anthony Traboulsee, David K.B. Li, Amit Bar-Or, Frederik Barkhof, Xavier Montalban, David Leppert, Anna Baldinotti, Hans-Martin Schneble, Harold Koendgen, Annette Sauter, Qing Wang, Stephen L. Hauser**

**Corresponding author:
Prof. Ludwig Kappos, MD
Research Center for Clinical Neuroimmunology and Neuroscience Basel (RC2NB)
Departments of Head, Spine and Neuromedicine, Clinical Research, Biomedicine and Clinical Research,
University Hospital Basel
University of Basel, Basel
Switzerland
Email: ludwig.kappos@usb.ch**


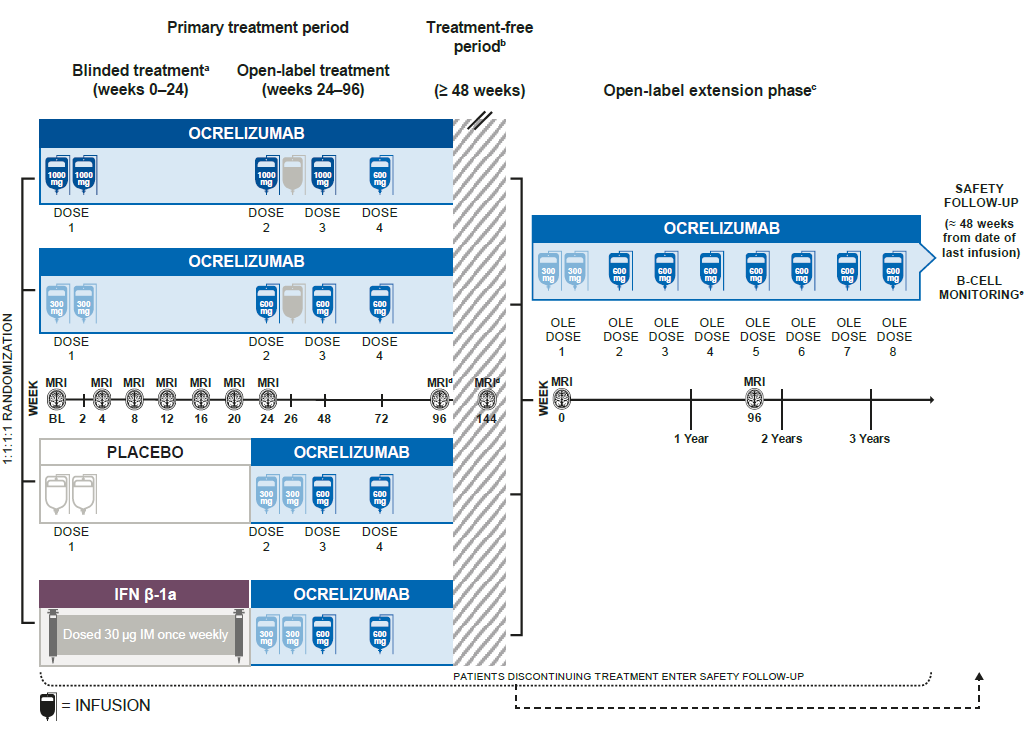


**Supplementary Fig. 1** NCT00676715 study design

First patient entered the study on July 17, 2008 and the clinical cutoff date was January 3, 2020. The median (IQR) overall study duration was 237.6 weeks (146.4–557.0)

^a^Double-blind treatment for PBO and ocrelizumab groups; rater-blinded for IFN β-1a

^b^Variable duration comprising post-treatment follow-up of at least 48 weeks (all participants) and a subsequent treatment-free interval without assessment prior to OLE commencement. B-cell monitoring was performed in patients who did not enter the OLE. See main text for details

^c^OLE was not mandatory; in the OLE, a baseline MRI was performed in all participants who did not have a final (week 144) MRI assessment in the assessed TFP

^d^MRI at weeks 96 and 144 were only performed in patients initially randomized to ocrelizumab

^e^Continued monitoring occurs if B cells are not repleted
 *Assessed TFP* assessed treatment-free period, *BL* baseline, *IFN* interferon, *IM* intramuscular, *IQR* interquartile range, *OLE* open-label extension, *PBO* placebo
