# Supplementary material for: Ocrelizumab exposure in relapsing–remitting multiple sclerosis: 10-year analysis of the phase 2 randomized clinical trial and its extension
Source: J Neurol. 2023 Oct 31;271(2):642–57. doi: 10.1007/s00415-023-11943-4 (PMC10827899; doi:10.1007/s00415-023-11943-4)
Supplement: Supplementary file 12 — Supplementary file12 (DOCX 140 KB) [file 415_2023_11943_MOESM12_ESM.pdf]

Ocrelizumab exposure in relapsing–remitting multiple sclerosis: 10-year analysis of the phase 2 randomized clinical trial and its extension

Journal of Neurology

Authors: Ludwig Kappos, Anthony Traboulsee, David K.B. Li, Amit Bar-Or, Frederik Barkhof, Xavier Montalban, David Leppert, Anna Baldinotti, Hans-Martin Schneble, Harold Koendgen, Annette Sauter, Qing Wang, Stephen L. Hauser

Corresponding author:
Prof. Ludwig Kappos, MD
Research Center for Clinical Neuroimmunology and Neuroscience Basel (RC2NB)
Departments of Head, Spine and Neuromedicine, Clinical Research, Biomedicine and Clinical Research, University Hospital Basel
University of Basel, Basel
Switzerland
Email: ludwig.kappos@usb.ch

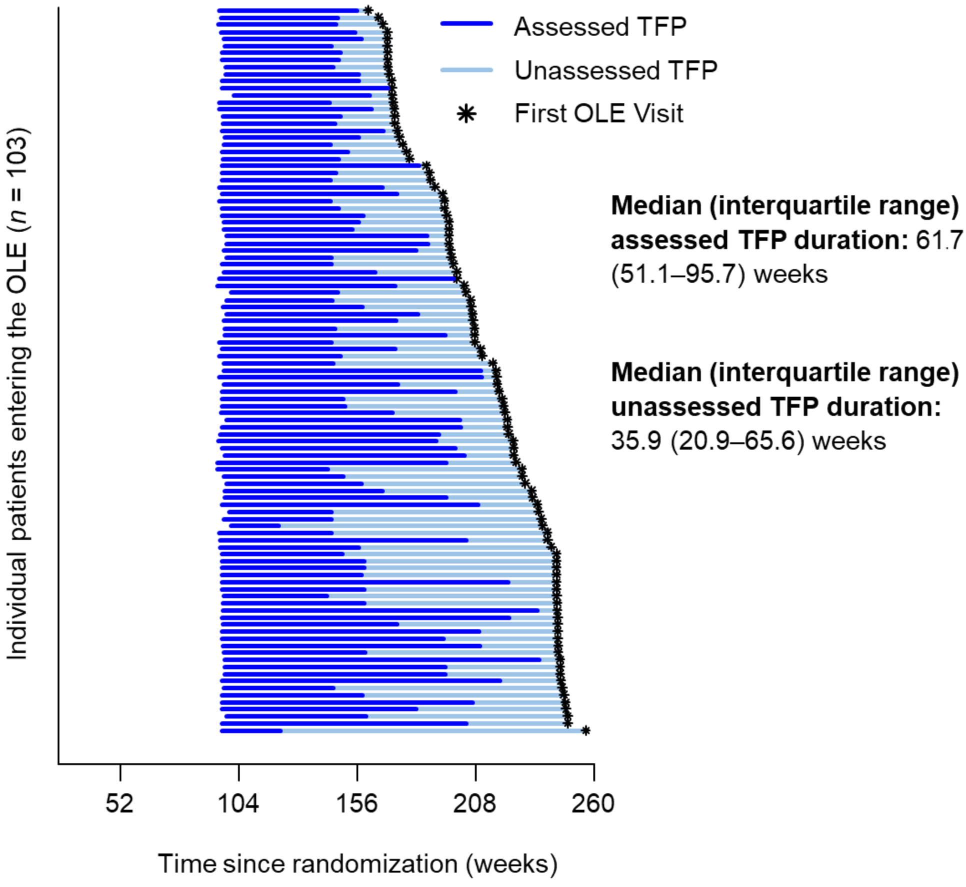

Median (interquartile range) assessed TFP duration: 61.7 (51.1–95.7) weeks

Median (interquartile range) unassessed TFP duration: 35.9 (20.9–65.6) weeks

Supplementary Fig. 2 Duration of the study treatment-free period for each patient who entered the OLE to receive ocrelizumab 600 mg every 24 weeks (n = 103)

Assessed TFP assessed treatment-free period, *IQR* interquartile range, *OLE* open-label extension, *unassessed TFP* unassessed treatment-free period
